# HDAC8 inhibition targets STAT3–MYC axis and synergizes with Venetoclax in KMT2A-rearranged acute myeloid leukemia

**DOI:** 10.1038/s41375-026-02950-1

**Published:** 2026-04-21

**Authors:** Lianjun Zhang, Wancheng Guo, Yu-Hsuan Fu, Dijiong Wu, Man Li, Ying-Chieh Chen, Chi-Yang Tseng, Wei-Kai Hua, Le Xuan Truong Nguyen, Xin He, Haojie Dong, Lei Zhang, Bin Zhang, Ling Li, Guido Marcucci, Ya-Huei Kuo

**Affiliations:** 1 Department of Hematological Malignancies Translational Science, Gehr Family Center for Leukemia Research, Beckman Research Institute of City of Hope, Duarte, CA USA; 2 Irell and Manella Graduate School of Biological Sciences, Beckman Research Institute of the City of Hope, Duarte, CA USA; 3 The First Affiliated Hospital of Zhejiang Chinese Medical University, Zhejiang Provincial Hospital of Chinese Medicine, Hangzhou, China

**Keywords:** Targeted therapies, Acute myeloid leukaemia

## Abstract

KMT2A-rearranged (KMT2A-r) acute myeloid leukemia (AML) is an aggressive AML subtype characterized by 11q23 chromosomal rearrangements involving *KMT2A* gene and clinically associated with poor prognosis. Herein, we show that *HDAC8* is upregulated in KMT2A-r AML and high HDAC8 is associated with poor overall survival in KMT2A-r AML patients. Using a *KMT2A::MLLT3* mouse model, we demonstrate that both genetic knockout and pharmacological inhibition of HDAC8 significantly delayed leukemia progression, prolonged survival and reduced disease recurrence. Mechanistically, HDAC8 inhibition downregulates STAT3–MYC axis independent of *TP53* status across AML genetic subtypes. Biochemical assays revealed that HDAC8 binds directly to STAT3, promoting its deacetylation and stabilization, while HDAC8-selective inhibitor (HDAC8i) treatment results in increased STAT3 acetylation and subsequent STAT3 degradation which in turn downregulates MYC. Given that STAT3–MYC signaling promotes cell survival and Venetoclax resistance, we show that HDAC8i exhibits synergistic anti-leukemia activity with Venetoclax in primary AML cells regardless of *TP53* status. Combination of HDAC8i and Venetoclax synergistically reduced leukemia burden and significantly prolonged survival in both *KMT2A::MLLT3* AML and patient-derived xenograft models. This study highlights the regulatory function of HDAC8 on STAT3–MYC and provides the proof-of-principle for targeting HDAC8 in combination with Venetoclax for the treatment of KMT2A-r AML.

## Introduction

Acute myeloid leukemia (AML) is a malignant hematologic disorder characterized by the uncontrolled proliferation of abnormal progenitor cells, resulting in the accumulation of immature myeloid blasts in the bone marrow (BM) and peripheral blood (PB). Despite advances in chemotherapy and hematopoietic stem cell transplantation, AML remains difficult to treat, particularly in patients with high-risk genetic alterations [1, 2]. KMT2A-rearranged (KMT2A-r) AML, defined by 11q23 chromosomal translocation involving the *KMT2A* (*MLL*) gene and fusion partners such as *MLLT3* (*AF9)*, *MLLT1* (*ENL)*, and *AFF1* (*AF4)*, represents a clinically aggressive disease subtype with poor prognosis [3]. KMT2A-r AML is especially prevalent in infant leukemia and is associated with therapy resistance, high relapse rates, posing a significant clinical challenge [4].

Venetoclax, a selective BCL-2 inhibitor approved for older or unfit AML patients, has demonstrated significant initial therapeutic responses but is often limited by the rapid emergence of resistance and disease relapse [5]. These challenges are particularly evident in high-risk AML subtypes with complex genetic and epigenetic abnormalities that activate alternative anti-apoptotic pathways, including MCL-1 and BCL-XL [6]. Aberrant activation of STAT3 and MYC signaling has also been implicated in Venetoclax resistance by promoting cell survival and proliferation [7, 8]. Consequently, Venetoclax-based regimens often fail to achieve durable responses.

Histone deacetylase 8 (HDAC8), a class I histone deacetylase, regulates chromatin remodeling and gene expression through the deacetylation of histone and non-histone proteins, thereby modulating key cellular processes such as cell cycle progression, differentiation, and apoptosis [9]. HDAC8 has emerged as a potential therapeutic target in multiple malignancies, including breast cancer, melanoma, and leukemia [10–13]. Previous studies have shown that HDAC8 inhibition enhances p53 acetylation and activation, selectively targets inv(16) AML leukemia stem cells (LSC) [14] and suppresses leukemia progression in FLT3-ITD^+^ AML [15]. Notably, HDAC8 inhibition reportedly induces cell cycle arrest and modulates canonical WNT signaling in AML cell lines lacking p53 [16], indicating that HDAC8 promotes AML through both p53-dependent and p53-independent mechanisms.

Here, we investigate the role of HDAC8 in KMT2A-r AML and identify a novel mechanism whereby HDAC8 interacts with STAT3 to regulate the STAT3–MYC signaling axis independently of p53. Furthermore, we demonstrate the synergistic anti-leukemia activity and therapeutic potential of combining an HDAC8 inhibitor with Venetoclax in KMT2A-r AML.

## Methods

### Mice

*Hdac8*^*flox/flox*^ [*Hdac8*^*f/f(y)*^] mice were crossed with *Mx1-Cre* transgenic mice to generate *Mx1-Cre/Hdac8*^*flox/flox*^ conditional knockout mice, which were backcrossed to the C57BL/6 (Jackson Laboratory, #000664) background for more than 10 generations [14, 17]. Mice (6–10 weeks old) were induced with polyinosinic–polycytidylic acid [poly (I:C)] (InvivoGen) at 14 mg/kg/dose every other day for 7 doses. Transplantation of BM was performed using 6–10 weeks old wild-type (WT) C57BL/6 mice. All mice were maintained in an Association for Assessment and Accreditation of Laboratory Animal Care–accredited animal facility. All experimental procedures were performed following federal and state government guidelines and established institutional guidelines and protocols approved by the Institutional Animal Care and Use Committee (IACUC #07046) at the City of Hope (COH).

### Human samples

Primary AML samples were obtained from patients treated at COH (Table S1) and controls were from healthy donor’s BM or mobilized PB stem cells. Samples were acquired with signed informed consent under procedures and protocols approved by COH Institutional Review Board (IRB #06229, 03162, 07047, 18067), under an assurance filed with and approved by the Department of Health Services. CD34^+^ cells were enriched using EasySep Human CD34 Positive Selection Kit II (StemCell Technologies).

### Drug synergy analysis

The synergistic effect of drug combination was assessed using SynergyFinder (https://synergyfinder.aittokallio.group/202602072043365112/) [18]. Drug dose–response curves were fitted using a four-parameter log-logistic model, and half-maximal inhibitory concentrations (IC_50_) were calculated using the drc package in R. The Highest Single Agent (HSA) method was employed to calculate the combination index, referred to as the Synergy Score. Specifically, Synergy Scores were interpreted as follows: >10, synergistic interaction; −10 to 10, additive interaction; and <−10, antagonistic interaction between the two drugs.

### Statistical analysis

Flow cytometry data were analyzed using FlowJo (10.8.1). Plots and statistical analyses were performed using R (version 4.3.1 and 4.4.2) or GraphPad Prism (10.2.0). Experimental data are presented as mean ± standard error of the mean (mean ± SEM). Between-group comparisons were conducted using unpaired Student’s *t* test, Wilcoxon rank-sum test, and Log-rank (Mantel–Cox) test for survival, and p-values < 0.05 were considered statistically significant (*p < 0.05; **p < 0.01; ***p < 0.001).

Additional methods are described in the supplementary methods.

## Results

### HDAC8 upregulation driven by SOX4 promotes KMT2A-r AML progression

**Fig. 1:**
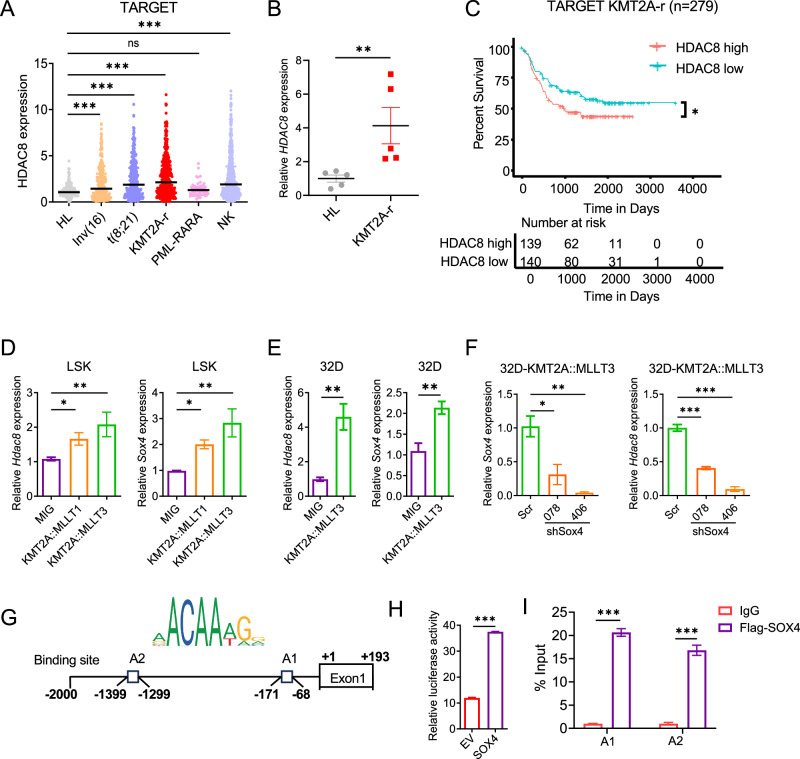
HDAC8 upregulation driven by SOX4 contributes to KMT2A-r AML Progression. **A** *HDAC8* mRNA expression levels as transcript per million (TPM) across different AML genetic subtypes, including inv(16) (*n* = 219), t(8;21) (*n* = 274), KMT2A-r (*n* = 431), PML-RARA (*n* = 56), normal karyotype (NK; *n* = 504) and healthy (HL) controls (*n* = 246) in the TARGET AML database. Horizontal lines indicate mean values; Statistical significance was assessed using Dunn’s multiple comparisons test (***p < 0.001). **B** Relative *HDAC8* mRNA levels assessed by qPCR analysis in CD34^+^ KMT2A-r AML (*n* = 5) and HL (*n* = 5). *ACTB* was used as an internal control. Data are presented as mean ± SEM; Statistical significance was assessed using an unpaired test (**p < 0.01). **C** Overall survival curve for KMT2A-r AML patients with HDAC8-high (*n* = 139) vs. HDAC8-low (*n* = 140) expression determined using a median cutoff. Statistical significance was assessed using a log-rank (Mantel–Cox) test (*p < 0.05). **D** Relative mRNA expression level for *Hdac8* and *Sox4* assessed by qPCR in murine LSK cells transduced with MIG, *KMT2A::MLLT3* or *KMT2A::MLLT1* (*n* = 3). *B2m* was used as an internal control. Data are presented as mean ± SEM; Statistical significance was assessed using an unpaired test (*p < 0.05; **p < 0.01). **E** Relative *Hdac8* and *Sox4* mRNA levels assessed by qPCR in 32D cells transduced with the MIG or *KMT2A::MLLT3* (*n* = 3). *B2m* was used as an internal control. Data are presented as mean ± SEM; Statistical significance was assessed using an unpaired test (**p < 0.01). **F** Relative *Sox4* and *Hdac8* mRNA levels in 32D-*KMT2A::MLLT3* cells expressing scrambled control (Scr) or shRNA-targeting Sox4 (shSox4-078 or 406) (*n* = 3). *B2m* was used as an internal control. Data are presented as mean ± SEM; Statistical significance was assessed using unpaired test (*p < 0.05; **p < 0.01; ***p < 0.001). **G** SOX4 binding sites within the *HDAC8* promoter region (-2000 bp to +193 bp) predicted by JASPAR (A1, A2). **H** Bar plot of luciferase activity normalized to Renilla (*n* = 3). 293T cells were co-transfected with HDAC8-pGL3 (–2000 to +193 bp) and either empty vector (EV) or SOX4 plasmid. **I** ChIP assay with anti-Flag or Rabbit IgG antibody in 293T cells expressing Flag-tagged-SOX4 followed by qPCR using two sets of primers spanning putative binding sites A1 (-171 to -68) or A2 (–1399 to –1299). Results are calculated as % Input (*n* = 3). Data are presented as mean ± SEM; Statistical significance was assessed using an unpaired test (***p < 0.001).

To assess the relevance of HDAC8 across AML subtypes, we analyzed its expression in the Therapeutically Applicable Research to Generate Effective Treatments (TARGET) AML RNA sequencing (RNA-seq) dataset [19, 20]. *HDAC8* expression was significantly higher in inv(16), t(8;21), KMT2A-r and normal karyotype (NK) AML compared with healthy (HL) donors (Fig. 1A). Quantitative (q)PCR assays confirmed elevated *HDAC8* expression in CD34^+^ cells from KMT2A-r AML patients (Table S1) relative to HL donors (Fig. 1B). Survival analysis of the TARGET AML cohort revealed that high *HDAC8* expression (median cutoff) was associated with significantly poorer overall survival in KMT2A-r AML patients (*n* = 279; *p* = 0.024) (Fig. 1C). These findings suggest a potential disease-promoting role of HDAC8 in KMT2A-r AML.

*HDAC8* transcription is reportedly activated by *SOX4* in adult T-cell leukemia/lymphoma [21]. Consistently, correlation analysis across four independent AML RNA-seq datasets (GSE131184, GSE114868, GSE76008, GSE68172) demonstrated a significant positive correlation between *SOX4* and *HDAC8* expression (Fig. S1A). We therefore hypothesized that *HDAC8* upregulation in KMT2A-r AML may be driven by *SOX4*. To test this, murine lineage^-^Sca1^+^ckit^+^ (LSK) stem and progenitor cells were transduced with an MSCV-IRES-GFP (MIG) vector expressing either *KMT2A::MLLT1* or *KMT2A::MLLT3*, which resulted in concomitant upregulation of *Sox4* and *Hdac8* expression (Fig. 1D). Similarly, transduction of 32D cells with *KMT2A::MLLT3* significantly increased *Sox4* and *Hdac8* expression (Fig. 1E). Moreover, *Sox4* knockdown in *KMT2A::MLLT3*–transduced 32D cells led to a marked reduction in *Hdac8* expression (Fig. 1F), confirming *Sox4* as a key transcriptional regulator of *Hdac8* downstream of KMT2A fusion proteins.

Using the transcription factor binding profile database JASPAR [22], we identified multiple potential *SOX4* binding sites within the *HDAC8* promoter region (Figs. 1G and S1B). To determine whether *SOX4* directly regulates *HDAC8* transcription, we generated an *HDAC8* promoter-driven luciferase reporter construct (HDAC8-pGL3) containing the HDAC8 promoter region spanning from -2000 to +193 bp relative to the transcription start site (Ensembl GRCh38.p14) (Fig. 1G). Co-transfection of SOX4 expression vector (SOX4-pcDNA3.1) and HDAC8-pGL3 reporter resulted in significantly increased luciferase activity compared with the empty vector (EV; Fig. 1H), indicating direct transcriptional activation of *HDAC8* by *SOX4*. Consistently, chromatin immunoprecipitation (ChIP) followed by qPCR analysis revealed significant enrichment of SOX4 at both distal (-1399 to -1299) and proximal (-171 to -68) binding sites within the *HDAC8* promoter compared with IgG controls (Fig. 1I).

### Genetic and pharmacological inhibition of HDAC8 reduces KMT2A::MLLT3 AML burden

**Fig. 2:**
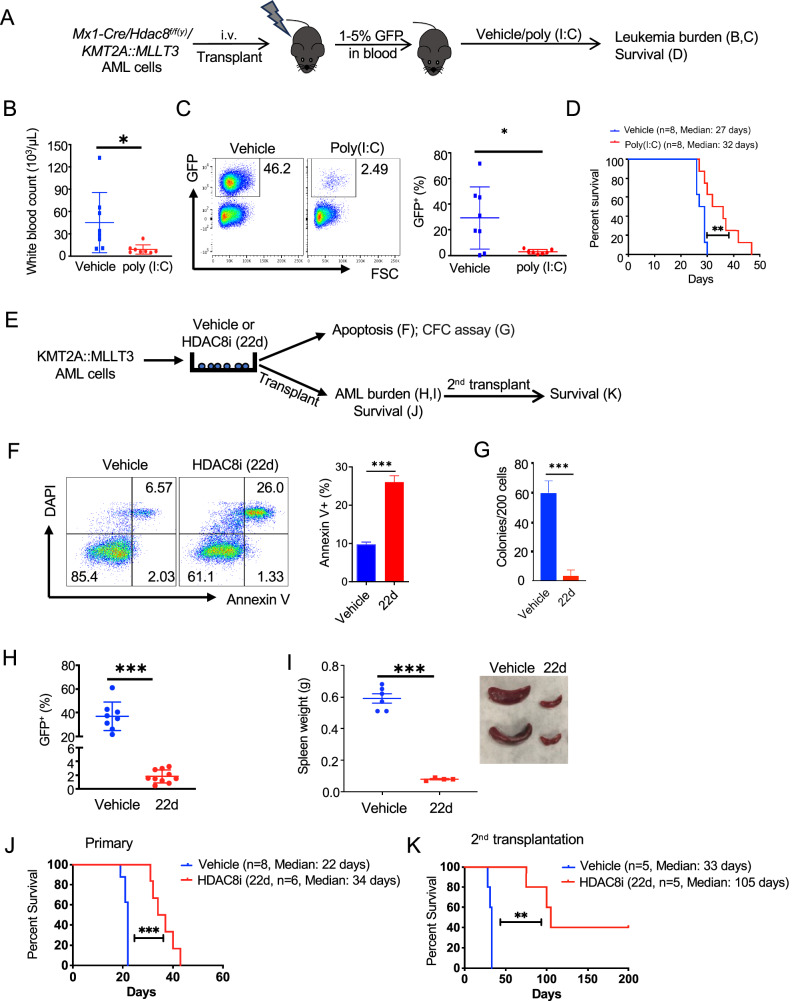
Genetic and pharmacological inhibition of HDAC8 reduces KMT2A::MLLT3 AML burden. **A** Schematic of the experimental strategy. LSK cells from a conditional *Hdac8*-deletion [*Mx1-Cre/Hdac8*^*f/f(y)*^] mice was transduced with an MIG-*KMT2A::MLLT3* (GFP^+^) vector, and sorted GFP^+^ cells were intravenously (i.v.) transplanted into C57BL/6 WT mice. Upon disease progression, mice were randomized into two groups followed by PBS vehicle control or poly (I:C) injection to induce *Hdac8* deletion. **B** White blood cell counts in vehicle control (*n* = 8) and poly (I:C)-treated (*n* = 8) groups at 3 weeks after treatment. Each dot represents data from an individual mouse; Data are presented as mean ± SEM; Statistical significance was assessed using an unpaired test (*p < 0.05) **C** (Left) Representative flow cytometric plots of GFP^+^ AML cells in PB at 3 weeks after treatment. (Right) Percentage of GFP^+^ AML cells in PB at 3 weeks after treatment (*n* = 8 for each group). Each dot represents data from an individual mice; Data are presented as mean ± SEM; Statistical significance was assessed using an unpaired test (*p < 0.05). **D** Kaplan–Meier survival curves (in days post-treatment) of mice in vehicle (*n* = 8) or poly (I: C)-treated groups (*n* = 8). Statistical significance was assessed using a log-rank (Mantel–Cox) test (**p < 0.01). **E** Schematic of experimental design. KMT2A::MLLT3 AML cells were treated with either HDAC8i (22d) or vehicle control, followed by assessment of apoptosis, colony-forming cell (CFC) assays and serial transplantation into WT C57BL/6 mice. **F** (Left) Representative Annexin V/DAPI staining flow cytometry plot in KMT2A::MLLT3 AML cells treated with vehicle or HDAC8i (22d, 10 μM) for 48 h. (Right) Apoptosis defined by Annexin V^+^ (%) in KMT2A::MLLT3 AML cells treated with vehicle (*n* = 3) or HDAC8i (22d, 10 μM) (*n* = 3) for 48 h. Data are presented as mean ± SEM; Statistical significance was assessed using an unpaired test (**p < 0.01). **G** Number of colonies (per 200 cells input) in vehicle- (*n* = 3) and HDAC8i (22d)-treated (*n* = 3) KMT2A::MLLT3 AML cells. Data are presented as mean ± SEM; Statistical significance was assessed using an unpaired test (***p < 0.001). **H** AML burden in PB accessed by GFP^+^(%) in mice receiving cells from vehicle (*n* = 8) or HDAC8i (22d)-treated (*n* = 10) at 2 weeks post-transplantation. Each dot represents data from an individual mouse; Data are presented as mean ± SEM; Statistical significance was assessed using an unpaired test (***p < 0.001). **I** Spleen weight (left) and representative spleen images (right) for vehicle- (*n* = 6) or HDAC8i (22d) (*n* = 4)-treated mice. Each dot represents data from an individual mouse; Data are presented as mean ± SEM; Statistical significance was assessed using an unpaired test (***p < 0.001). **J** Primary survival curve for recipients of vehicle- (*n* = 8; median survival 22 days) or HDAC8i (22 d)-treated (*n* = 6; median survival 34 days) mice. Statistical significance was assessed using a log-rank (Mantel–Cox) test (***p < 0.001). **K** Kaplan-Meier survival curve for secondary transplantation recipients for vehicle- (*n* = 5; median survival 33 days) or HDAC8i (22d)-treated (*n* = 5; median survival 105 days) group. Statistical significance was assessed using a log-rank (Mantel–Cox) test (**p < 0.01).

To determine the functional contribution of *HDAC8* to KMT2A-r AML progression, LSK cells isolated from conditional *Hdac8*-floxed [*Mx1-Cre/Hdac8*^*f/f(y)*^] mice were transduced with MIG-*KMT2A::MLLT3* (GFP^+^) and transplanted into wild-type (WT) mice. Upon detection of GFP^+^ leukemic cells in PB (1-5%), mice were randomly assigned to receive either poly (I:C) to induce Cre-mediated *Hdac8* deletion or PBS as a vehicle control (Fig. 2A). Poly (I:C) treated mice exhibited significantly reduced white blood cell counts and lower frequencies of circulating GFP^+^ leukemic cells compared with controls analyzed 3 weeks post-treatment (Fig. 2B, C). Importantly, the poly (I:C)-treated group had significantly prolonged survival relative to the control group (Fig. 2D).

Next, we evaluated the effects of pharmacological HDAC8 inhibition on *KMT2A::MLLT3* AML cells using a selective HDAC8 inhibitor (HDAC8i, 22d) [23] (Fig. 2E). Treatment with HDAC8i (22d, 10 μM) significantly increased apoptotic cell death (Fig. 2F) and markedly reduced the colony-forming capacity of *KMT2A::MLLT3* AML cells (Fig. 2G). To further evaluate the impact on AML progression, *KMT2A::MLLT3* AML cells were treated with either HDAC8i (22d) or vehicle, followed by transplantation into WT mice (Fig. 2E). Mice receiving HDAC8i (22d)-treated cells exhibited a significantly lower AML burden in PB (Fig. 2H) and reduced splenomegaly (Fig. 2I) compared with vehicle-treated controls. Survival analysis revealed that primary recipients of HDAC8i (22d)-treated cells (*n* = 6) had significantly prolonged survival compared with recipients of vehicle-treated cells (*n* = 8) (median: 34 vs. 22 days, *p* = 0.0041) (Fig. 2J). To assess the leukemia-initiating potential of the remaining cells, secondary transplantation was performed using BM cells isolated from moribund primary recipients. Notably, mice receiving cells from the HDAC8i (22d)-treated group (*n* = 5) exhibited a lower incidence of disease recurrence (60% vs. 100%) and significantly extended survival compared with recipients of vehicle-treated group (*n* = 5) (median: 105 vs. 33 days, *p* = 0.0034) (Fig. 2K). Altogether, these results demonstrate that SOX4-driven upregulation of HDAC8 plays a critical role in KMT2A-r AML progression. Both genetic deletion and pharmacological inhibition of HDAC8 significantly reduce leukemic burden, impair leukemia-initiating capacity, and delay disease recurrence, highlighting HDAC8 as a promising therapeutic target in KMT2A-r AML.

### HDAC8 inhibition targets STAT3/MYC axis independent of *TP53* status

**Fig. 3:**
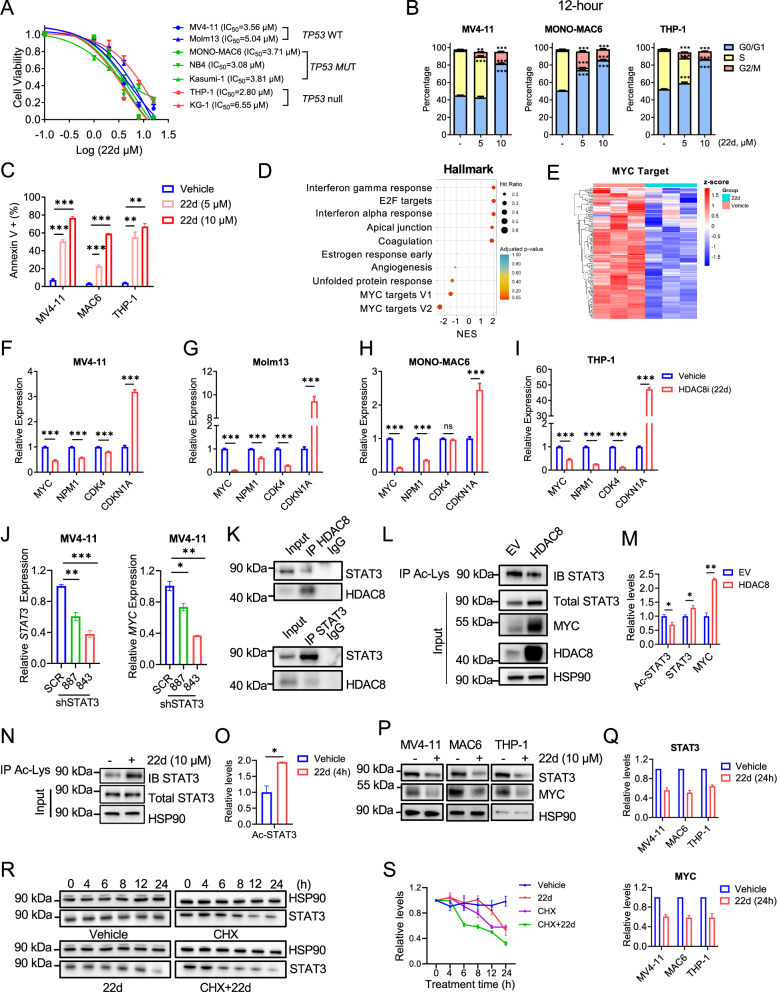
HDAC8 inhibition targets the STAT3/MYC axis independent of *TP53* status. **A** Cell viability inhibitory curve and IC_50_ of HDAC8i (22d) in AML cell lines with *TP53*-WT (MV4-11, Molm-13), *TP53*-MUT (MONO-MAC6, NB4, Kasumi-1) or *TP53*-null (THP-1, KG-1) (*n* = 3). **B** Cell cycle analysis assessed by EdU-PI dual staining in MV4-11, MONO-MAC6 and THP-1 cells treated with vehicle or HDAC8i (22d, 5 μM or 10 μM) for 12 h (*n* = 3). Data are presented as mean ± SEM; Statistical significance was assessed using an unpaired test (**p < 0.01; ***p < 0.001). **C** The percentage of Annexin V^+^ apoptotic cells in KMT2A-r AML cell lines (MONO-MAC6, MV4-11, THP-1) treated with HDAC8i (22d, 5 μM or 10 μM, 24 h) or vehicle control (*n* = 3). Data are presented as mean ± SEM; Statistical significance was assessed using an unpaired test (**p < 0.01; ***p < 0.001). **D** Gene set enrichment analysis (GSEA) analysis of RNA-seq data from MV4-11 cells treated with HDAC8i (22d, 10 μM, 12 h) versus vehicle control. Dot plot showing the top 5 positively enriched (highest NES) and top 5 negatively enriched (lowest NES) Hallmark pathways. Dot size represents the hit ratio, defined as the proportion of leading-edge genes in the gene set. The x-axis indicates normalized enrichment scores (NES), and colors denote p-values. **E** Heatmap showing gene-wise z-score–normalized expression of leading-edge Hallmark MYC targets (125 genes) in MV4-11 cells treated with vehicle and HDAC8i (22d), with red indicating high expression and blue indicating low expression. **F**–**I** Relative expression of *MYC, NPM1, CDK4, CDKNA1* assessed by qPCR analysis in MV4-11, Molm-13, MONO-MAC6 and THP-1 AML cells treated with vehicle or HDAC8i (22d, 10 μM, 24 h). *ACTB* was used as an internal control. Data are presented as mean ± SEM; Statistical significance was assessed using an unpaired test (***p < 0.001) (*n* = 3). **J** Relative *STAT3* and *MYC* expression in scrambled control (SCR) or STAT3knocked-down (shRNA-887 and 843) MV4-11 cells (*n* = 3). *ACTB* was used as an internal control. Data are presented as mean ± SEM; Statistical significance was assessed using an unpaired t-test (*p < 0.05; **p < 0.01; ***p < 0.001). **K** Co-immunoprecipitation (co-IP) using anti-HDAC8 (top) or anti-STAT3 (bottom) antibodies followed by immunoblotting (IB) to detect HDAC8 and STAT3 interaction in MONO-MAC6 cells (*n* = 3). Representative blot is shown; additional replicates are provided in the Supportive Information. **L** Representative IB of acetylated (Ac)-STAT3 and total STAT3, MYC in MV4-11 cells with HDAC8-overexpression (OE) or empty vector (EV; pCDH-EF1α-MCS-IRES-RFP) by co-IP with anti-acetylated-Lysine (Ac-Lys) antibody (*n* = 3). Representative blot is shown; additional replicates are provided in the Supportive Information. **M** Relative levels of Ac-STAT3 and total STAT3, MYC in MV4-11 cells with HDAC8-OE or EV were quantified from three independent experiments using Image J; Data are presented as mean ± SEM (*n* = 3); Statistical significance was assessed using unpaired t-test (*p < 0.05; **p < 0.01). **N** Co-IP with anti-Ac-Lys antibody to detect Ac-STAT3 in MONO-MAC6 cells treated with vehicle or HDAC8i (22d, 10 μM, 4 h) (*n* = 3). Representative blot is shown; additional replicates are provided in the Supportive Information. **O** Relative levels of Ac-STAT3 protein quantified from three independent experiments using Image J; Data are presented as mean ± SEM (*n* = 3); Statistical significance was assessed using an unpaired t-test (*p < 0.05). Representative immunoblotting (**P**) and quantification (**Q**) of STAT3 and MYC protein levels in AML cell lines (MV4-11, MONO-MAC6, THP-1) treated with vehicle or HDAC8i (22d, 10 μM, 24 h) (*n* = 3). Representative blot is shown; additional replicates are provided in the Supportive Information. **R** Western blot for STAT3 protein following treatment with vehicle, HDAC8i (22d, 10 μM), cycloheximide (CHX; 100 μM), or both HDAC8i (22d) and CHX at indicated time points (0, 4, 6, 8, 12, 24 h) in MONO-MAC6 cells (*n* = 3). Representative blot is shown; additional replicates are provided in the Supportive Information. **S** Line graphs represent mean STAT3 protein levels over time quantified from three independent experiments; Data are presented as mean ± SEM (*n* = 3).

*TP53* mutations are associated with poor prognosis and are frequently observed in therapy-related AML (up to 30-40%) and de novo AML with complex karyotype (up to 70–80%) [24]. Given the widespread upregulation of HDAC8 in AML, we sought to determine whether HDAC8 inhibition exerts anti-leukemic effects independent of *TP53* status. To this end, we evaluated a panel of AML lines representing diverse oncogenic drivers and *TP53* genotypes (Fig. S2A). HDAC8i (22d) treatment effectively reduced cell viability across all tested cell lines regardless of *TP53* status, with IC_50_ values ranging from 2.80 to 6.55 μM (Fig. 3A). Similar inhibitory effects were observed with PCI-34051 [25], a well-characterized and selective HDAC8 inhibitor (Fig. S3A). HDAC8i (22d) induced significant G_0_/G_1_ cell cycle arrest as early as 4-12 h (Figs. 3B and S2B), followed by increased apoptosis at 24 h (Figs. 3C and S2C) in both *TP53*-WT and *TP53*-mutant (MUT) AML cell lines. Consistently, immunoblot analysis revealed significant downregulation of key cell cycle regulators, including CDK4, CDK6, and cyclin D3, accompanied by increased levels of cleaved caspase-3 following HDAC8i (22d) treatment (Fig. S3B, C). These results indicate that HDAC8 inhibition suppresses AML cell proliferation and promotes apoptotic cell death independent of *TP53* status.

To elucidate the molecular mechanisms underlying these effects, we performed RNA-seq for AML cell lines MV4-11 (heterozygous *TP53*-P72R polymorphism), Kasumi-1 (homozygous *TP53*-R248Q) and KG-1 (*TP53-*null) following short-term treatment with HDAC8i (22d, 12 h) (Tables S6–8). Gene Set Enrichment Analysis (GSEA) [26, 27] of Hallmark gene sets revealed significant downregulation of “MYC Targets” across all three cell lines (Figs. 3D, E and S4; Table S9). Consistent with these findings, qPCR analysis confirmed that HDAC8i reduced expression of *MYC* and its downstream targets, while inducing expression of *CDKN1A* and pro-apoptotic genes *BBC3*, *PMAIP1*, *BNIP3* and *BNIP3L* across AML cell lines irrespective of *TP53* status (Figs. 3F–I and S5A, B). Notably, pharmacological inhibition of MYC using 10058-F4 similarly upregulated *CDKN1A* and induced pro-apoptotic gene expression in *TP53*-MUT and *TP53*-null AML cell lines (Fig. S6A–D). Moreover, genetic deletion of *Hdac8* in a murine *KMT2A::MLLT3* AML model recapitulated these transcriptional changes, with reduced expression of Myc target genes and increased expression of pro-apoptotic genes (Fig. S5C), indicating that HDAC8 directly regulates MYC-driven transcriptional programs, including *CDKN1A* and pro-apoptotic genes.

*MYC* is broadly overexpressed in AML and plays a central role in sustaining leukemic cell survival and proliferation. *STAT3* is a known upstream regulator of *MYC* transcription, and the STAT3–MYC axis has been implicated in LSC maintenance [28]. Consistent with this, *STAT3* knockdown significantly reduced *MYC* expression in MV4-11 AML cells (Fig. 3J). We therefore hypothesized that HDAC8 promotes AML cells survival by suppressing STAT3 acetylation, thereby stabilizing STAT3 and sustaining MYC expression. Co-immunoprecipitation (Co-IP) assays confirmed physical interaction between HDAC8 and STAT3 (Figs. 3K and S7A). HDAC8 overexpression (OE) resulted in reduced STAT3 acetylation concomitant with increased total STAT3 and MYC protein levels in MV4-11 cells (Fig. 3L, M). Conversely, HDAC8i (22d) treatment led to a rapid increase in STAT3 acetylation within 4 h (Figs. 3N, O and S7B, C), followed by a pronounced reduction in STAT3 and MYC protein levels at 24 h (Fig. 3P, Q) in multiple KMT2A-r AML cell lines (MV4-11, MONO-MAC6 and THP-1) as well as in non-KMT2A-r AML cell lines (Kasumi-1, KG-1; Fig. S7D, E). To directly assess the impact of HDAC8i (22d) on STAT3 protein stability, we performed cycloheximide (CHX) chase experiments. STAT3 degradation was markedly accelerated in cells treated with CHX plus HDAC8i (22d) compared with CHX alone, with a substantial reduction of STAT3 protein levels evident by 6 h (Fig. 3R, S). Collectively, these findings demonstrate that HDAC8 promotes STAT3 stability by suppressing STAT3 acetylation, thereby sustaining MYC expression and AML survival. Inhibition of HDAC8 disrupts this STAT3–MYC axis by increasing STAT3 acetylation, promoting STAT3 degradation, and suppressing MYC-driven transcriptional programs in a *TP53*-independent manner.

### Anti-leukemic activity of HDAC8i (22d) and synergism with Venetoclax in AML

**Fig. 4:**
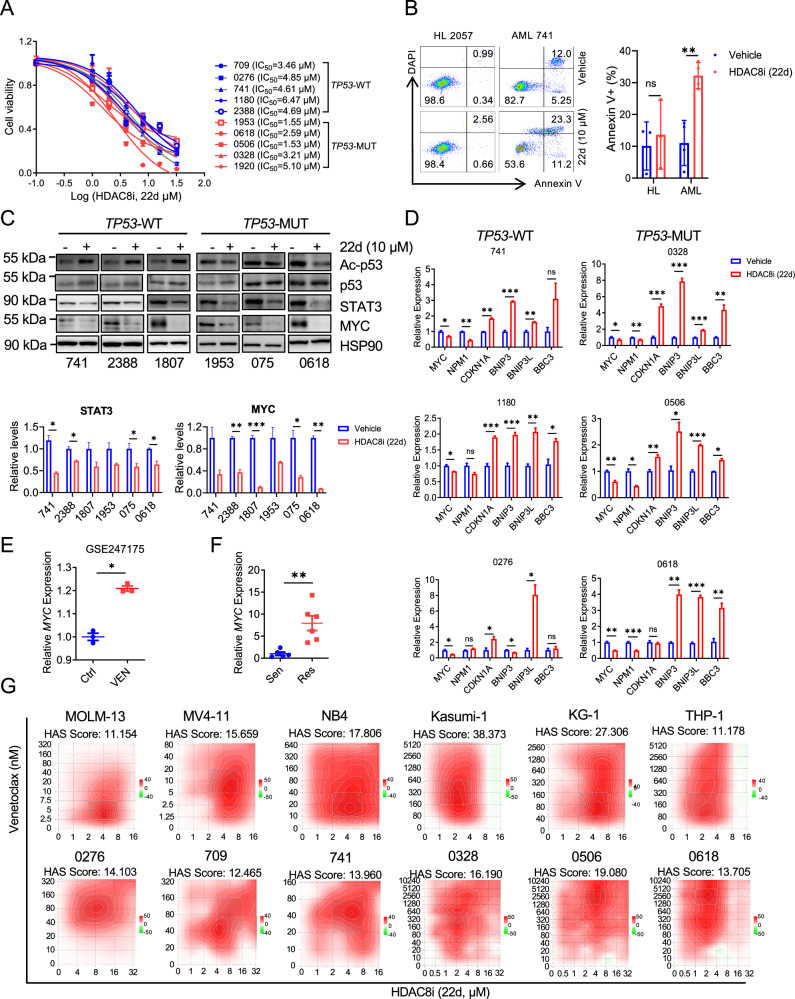
Synergistic anti-leukemic activity of HDAC8i (22d) and venetoclax in primary AML samples. **A** Cell viability curves and IC_50_ of HDAC8i (22d) in *TP53*WT (*n* = 5, blue) or *TP53*-MUT (*n* = 5, red) primary AML samples. Cell viability was measured using the CellTiter assay. **B** (Left) Representative flow cytometry plots showing Annexin V/DAPI staining in healthy (HL 2057) or AML patient (AML 741) samples treated with vehicle or HDAC8i (22d, 10 μM, 48 h). (Right) Bar plots show mean percentage of Annexin V^+^ cells from healthy (*n* = 3) or AML patient (*n* = 4) samples; Data are presented as mean ± SEM; Statistical significance was assessed using an unpaired t-test (**p < 0.01). **C** (Top) Representative blot of Ac-p53, total p53, STAT3, MYC in *TP53*-WT (left) or *TP53*-MUT (right) primary AML samples treated with vehicle or HDAC8i (22d, 10 μM, 24 h). HSP90 was used as a loading control (*n* = 3). Representative blot is shown; additional replicates are provided in the Supportive Information. (Bottom) Relative levels of STAT3, MYC protein quantified from three independent experiments using Image J; Data are presented as mean ± SEM (*n* = 3); Statistical significance was assessed using  an unpaired t-test (*p < 0.05; **p < 0.01; ***p < 0.001). **D** Relative mRNA expression levels for *MYC*, *NPM1*, *CDKN1A, BNIP3*, *BNIP3L* and *BBC3* assessed by qPCR in *TP53*-WT (left; samples 741,1180, 0276) or *TP53*-MUT (right; samples 0328, 0506, 0618) primary AML cells treated with vehicle or HDAC8i (22d, 10 μM, 24 h). *ACTB* was used as an internal control. Data are presented as mean ± SEM; Statistical significance was assessed using an unpaired t-test (*p < 0.05; **p < 0.01; ***p < 0.001) (*n* = 3). **E** Relative *MYC* expression in MV4-11 cells treated with vehicle or Venetoclax (100 nM, 48 h) obtained from GEO dataset GSE247175 (*n* = 3). Data are presented as mean ± SEM; Statistical significance was assessed using an unpaired t-test (*p < 0.05). **F** Relative *MYC* expression level assessed by qPCR in Venetoclax-sensitive (Sen, *n* = 5) and Venetoclax-resistant (Res, *n* = 6) AML samples (see Table S1). *ACTB* was used as an internal control. Data are presented as mean ± SEM; Statistical significance was assessed using an unpaired t-test (**P < 0.01). **G** Synergistic effects of Venetoclax and HDAC8i (22d) in AML cell lines (top) and primary AML samples (bottom) calculated by SynergyFinder. Synergy Score > 10 indicates a significant synergistic effect. The color scale represents the strength of synergy, with red indicating stronger synergy and green indicating weaker or antagonistic interactions.

To evaluate the clinical relevance of HDAC8 inhibition, we assessed the anti-leukemia activity of HDAC8i (22d) in primary AML cells with diverse *TP53* genotypes (Table S1). Consistent with findings in AML cell lines, HDAC8i (22d) significantly reduced the survival of primary AML cells with IC_50_ values ranging from 1.53 to 6.47 μM, irrespective of *TP53* status (Fig. 4A). Importantly, HDAC8i (22d) exhibited minimal cytotoxicity toward healthy donor samples (Fig. 4B).

In both *TP53*-WT and *TP53*-MUT AML samples, treatment with HDAC8i (22d, 10 μM; 24 h) resulted in a consistent reduction in STAT3 and MYC protein levels, independent of p53 acetylation status or total p53 expression (Figs. 4C and S8A). Concordantly, qPCR analysis showed downregulation of *MYC* and its downstream target *NPM1*, alongside upregulation of pro-apoptotic genes *BNIP3*, *BNIP3L* and *BBC3*, and cell cycle inhibitor *CDKN1A* in both *TP53*-WT and *TP53*-MUT AML samples (Figs. 4D and S8B).

STAT3 and MYC have been implicated in resistance to Venetoclax therapy in AML [8]. Analysis of publicly available RNA-seq data (GSE247175) revealed increased *MYC* expression in MV4-11 cells following Venetoclax treatment (Fig. 4E). Consistently, we observed higher *MYC* levels in Venetoclax-resistant (Res) samples compared with Venetoclax-sensitive (Sen) primary AML samples (Figs. 4F and S8C). Given our findings that HDAC8 inhibition suppresses STAT3–MYC axis, we hypothesized that combining HDAC8i (22d) with Venetoclax would exert synergistic anti-leukemia effects. To test this, we evaluated the combinatorial effects of HDAC8i (22d) and Venetoclax across multiple AML cell lines and primary AML samples. Drug interaction was quantified using SynergyFinder [18], with Highest Single Agent (HSA) scores greater than 10 defined as exhibiting synergy. Notably, robust synergistic interactions were observed across all AML cell lines and primary samples tested, regardless of *TP53* status (Figs. 4G and S9). These data support dual targeting of HDAC8 and BCL-2 as a promising therapeutic strategy to overcome Venetoclax resistance and enhance anti-leukemic efficacy in AML.

### Synergistic anti-leukemic activity of HDAC8i (22 d) and Venetoclax in AML preclinical mouse models

**Fig. 5:**
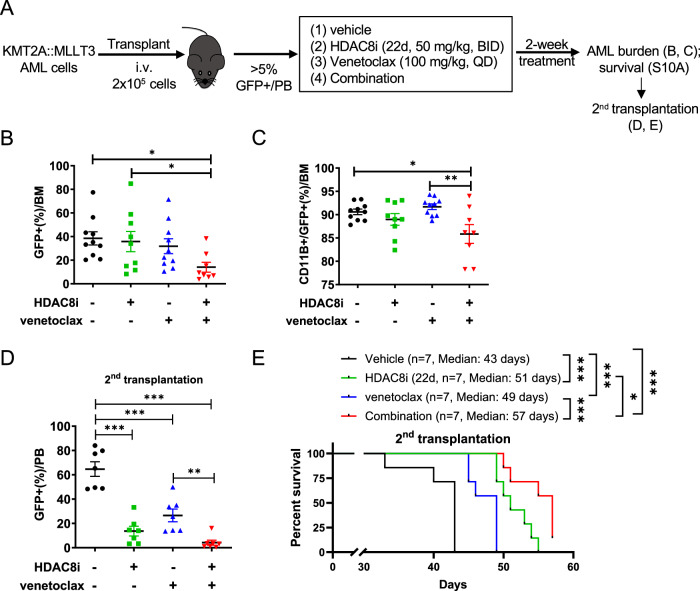
Anti-leukemic efficacy of HDAC8i (22d) and Venetoclax combination therapy in the *KMT2A::MLLT3* AML model. **A** Schematic of efficacy studies using the *KMT2A::MLLT3* AML model. *KMT2A::MLLT3* AML cells (GFP^+^, 2 × 10^5^/mouse) were intravenously transplanted into non-irradiated WT C57BL/6 mice. When GFP^+^ AML cells exceeded ~5% in the PB, mice were randomized and treated with vehicle, HDAC8i (22d, 50 mg/kg, BID, i.p. for 14 days), Venetoclax (100 mg/kg, QD, oral gavage for 14 days), or the combination of HDAC8i (22d) and Venetoclax (same dose/schedule as single agent) for two weeks. Following treatment, cohorts of mice were either monitored for survival or euthanized for assessment of AML burden, and BM cells from treated mice were subjected to secondary transplantation to evaluate leukemia-initiating capacity and survival. The percentage of GFP^+^ AML cells (**B**) and CD11B^+^/GFP^+^ cells (**C**) in the BM of mice in vehicle (*n* = 10), HDAC8i (22d, *n* = 9), Venetoclax (*n* = 10) or the combination (*n* = 8) treatment group. Each dot represents data from an individual mouse; data are presented as mean ± SEM; Statistical significance was assessed using one-way ANOVA (*p < 0.05; **p < 0.01). **D** The percentage of GFP^+^ AML cells in the PB of secondary transplantation recipients of vehicle (*n* = 7), HDAC8i (22d, *n* = 7), Venetoclax (*n* = 7) or combination (*n* = 7). Each dot represents data from an individual mouse; data are presented as mean ± SEM; Statistical significance was assessed using one-way ANOVA (**p < 0.01; ***p < 0.001). **E** Kaplan–Meier survival curves of secondary transplantation recipient mice receiving BM from vehicle (*n* = 7), HDAC8i (22d, *n* = 7), Venetoclax (*n* = 7) or the combination (*n* = 7) treatment. Statistical significance was assessed using a log-rank test (*p < 0.05; **p < 0.01; ***p < 0.001).

To further evaluate the therapeutic potential of the HDAC8i-Venetoclax combination in vivo, we employed a murine *KMT2A::MLLT3* AML model. *KMT2A::MLLT3* (GFP⁺) leukemia cells (2 × 10^5^) were transplanted into non-irradiated C57BL/6 WT mice, which were randomized and treated with either vehicle, HDAC8i (22d, 50 mg/kg, i.p., BID), Venetoclax (100 mg/kg, orally, QD), or the combination (same dose/schedule as single agent) for two weeks, once leukemia burden (GFP^+^) exceeded 5% in the PB (Fig. 5A). Combination therapy markedly reduced the frequency of GFP^⁺^CD11B^+^ leukemic cells in the BM compared with monotherapy (Fig. 5B, C). To evaluate the impact of leukemia-initiating capacity, we performed secondary transplantation of BM cells from treated mice right after the completion of treatment. Notably, recipients of BM cells from the combination treatment group showed markedly lower leukemia burden (Fig. 5D) and significantly improved survival (median: 57 days) compared with those receiving Venetoclax alone (median: 49 days) or vehicle control (median: 43 days) (Fig. 5E). These results suggest that concurrent HDAC8 and BCL-2 inhibition effectively targets leukemia-initiating LSCs.

We further examined the effects of HDAC8 inhibition and the combination therapy on immune cell populations. In the non-leukemic (GFP^-^) immune compartment, treatment with HDAC8i, Venetoclax or combination did not alter T cell frequencies but caused a modest yet significant reduction in B cells (B220^+^), accompanied by a reciprocal increase in myeloid cells (CD11b⁺, Gr1⁺) (Fig. S10A, B), consistent with lineage shifts observed in *Hdac8* conditional knockout mice [17]. Administration of HDAC8i alone (same dose and schedule) to normal WT mice caused no detectable alterations in PB counts or immune cell composition over time (up to 10 weeks). Venetoclax and combination treatment induced a modest lymphopenia, affecting both B and T cells (Fig. S11). Analysis of BM hematopoietic progenitor compartments (Fig. S12) and stromal populations (Fig. S13) revealed only limited myeloid expansion, indicating that HDAC8 inhibition largely spares normal hematopoiesis. These data demonstrate that the HDAC8i-Venetoclax combination preferentially targets leukemic cells, with minimal disruption of normal hematopoiesis, primarily driven by Venetoclax.

**Fig. 6:**
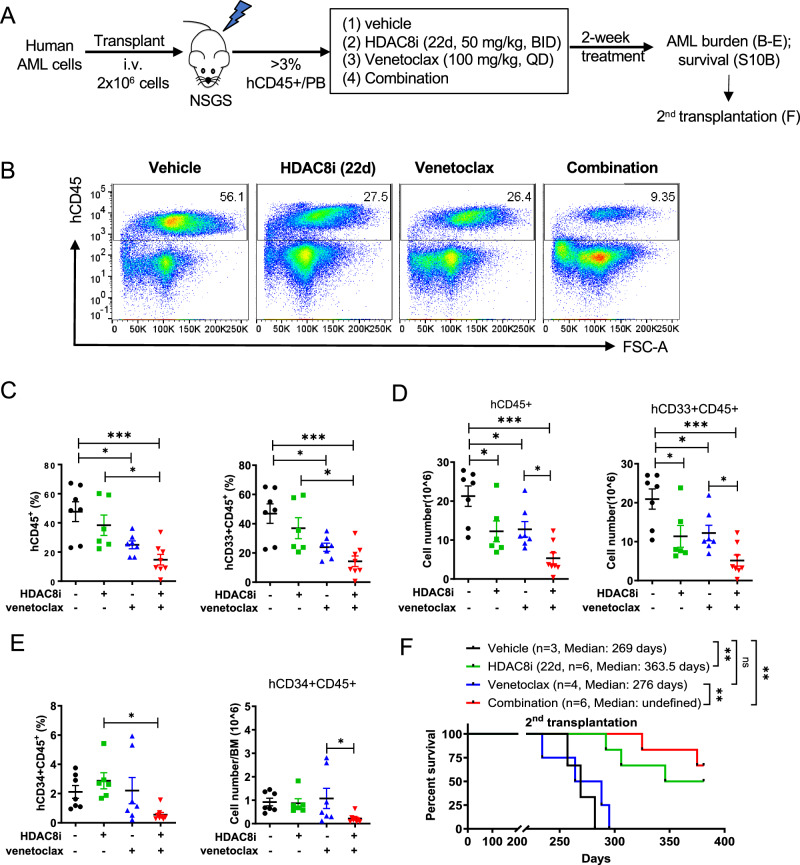
Anti-leukemic activity of HDAC8i (22d) and synergy with Venetoclax in KMT2A-r AML PDX model. **A** Schematic of efficacy studies using KMT2A-r AML PDX model. Primary human KMT2A-r AML (AML 741) cells (2 × 10^6^/mouse) were injected into irradiated (2 Gy) NSGS mice. Mice were randomized into treatment groups [vehicle, HDAC8i (22d, 50 mg/kg, BID, i.p. for 14 days), Venetoclax (100 mg/kg, QD, oral gavage for 14 days), combination therapy (same dose/schedule as single agent)] when hCD45^+^ cells in PB exceeded 3%. After two weeks of treatment, cohorts of mice were either monitored for survival or euthanized for assessment of AML burden, and secondary transplantation was performed to evaluate the long-term effect and impact on LSCs. **B** Representative flow cytometry plots of hCD45^+^ cells after treatment with vehicle, HDAC8i (22d), venetoclax or the combination. **C** The percentage (%) of hCD45^+^ (left) and hCD33^+^/CD45^+^ (right) cells in BM of vehicle (*n* = 7), HDAC8i (22d, *n* = 6), Venetoclax (*n* = 7) or the combination (*n* = 8) treated mice at the end of treatment. Each dot represents data from an individual mouse; data are presented as mean ± SEM; Statistical significance was assessed using one-way ANOVA (*p < 0.05; ***p < 0.001). **D** The total number of hCD45^+^ (left) and hCD33^+^/CD45^+^ (right) cells in BM of vehicle (*n* = 7), HDAC8i (22d, *n* = 6), Venetoclax (*n* = 7) or the combination (*n* = 8) treated mice at the end of treatment. Each dot represents data from an individual mouse; data are presented as mean ± SEM; Statistical significance was assessed using one-way ANOVA (*p < 0.05; ***p < 0.001). **E** The percentage (left) and total number (right) of hCD34^+^/CD45^+^ cells in the BM of vehicle (*n* = 7), HDAC8i (22d, *n* = 6), Venetoclax (*n* = 7) or the combination (*n* = 8) treated mice at the end of treatment. Each dot represents data from an individual mouse; data are presented as mean ± SEM; Statistical significance was assessed using one-way ANOVA (*p < 0.05). **F** Kaplan–Meier survival curves of secondary transplantation recipient mice from vehicle (*n* = 3), HDAC8i (22d, *n* = 6), Venetoclax (*n* = 4) or the combination (*n* = 6) treatment group. Statistical significance was assessed using a log-rank (Mantel–Cox) test (**p < 0.01).

We next evaluated the efficacy of the HDAC8i-Venetoclax combination in AML PDX models. Primary human AML cells (2 × 10^6^) were transplanted into sublethally irradiated (2 Gy) NGS/Tg (CMV-IL3, CSF2, KITLG) (NSGS) mice. Once leukemia burden reached ~3% of human (h)CD45^⁺^ AML in PB, mice were randomized to receive vehicle, HDAC8i (22d; 50 mg/kg, BID), Venetoclax (100 mg/kg, QD), or the combination (same dose/schedule as single agent) for 2 weeks (Fig. 6A). Combination therapy significantly reduced leukemic burden in the BM, as evidenced by decreased frequencies of hCD45^+^ and hCD33^+^CD45^+^ cells compared with monotherapy (Fig. 6B–D). Moreover, the proportion of hCD34^⁺^CD45^+^ cells was significantly reduced in the combination group (Fig. 6E), suggesting enhanced targeting of leukemic stem/progenitor cells. Consistently, secondary transplantation recipients from the combination-treated group survived significantly longer compared with those from monotherapy or vehicle-treated groups (Fig. 6F). Collectively, these in vivo findings demonstrate that concurrent inhibition of HDAC8 and BCL-2 synergistically enhances the therapeutic efficacy in AML, with preferential activity against LSCs while sparing normal hematopoiesis. These results provide strong preclinical support for the clinical development of HDAC8i-Velecoclax combination strategies in AML.

## Discussion

Our previous studies demonstrated that HDAC8 activity is enhanced in inv(16) AML through direct interaction with CBFβ::SMMHC fusion protein, resulting in increased HDAC8 stability, and a critical dependence on the HDAC8–p53 axis for leukemic cell survival [14, 29]. Additional work has shown that HDAC8 can be transcriptionally upregulated by FOXO1 and FOXO3 in response to tyrosine kinase inhibitor (TKI) treatment in FLT3-ITD^+^ AML and BRC-ABL^+^ B-cell acute lymphoblastic leukemia [15, 30]. In this study, we show that HDAC8 is broadly upregulated across multiple AML subtypes and is associated with poor prognosis in patients with KMT2A-r AML. Mechanistically, we identify SOX4 as a direct transcriptional regulator of HDAC8 in KMT2A-r AML, establishing a previously unrecognized oncogenic regulatory axis.

KMT2A fusion proteins have been shown to impair p53 function by suppressing its acetylation and transcriptional activity [31, 32], suggesting that HDAC8 may promote leukemogenesis through p53-independent mechanisms in this context. Nevertheless, HDAC8i treatment enhanced p53 acetylation in *TP53*-WT AML samples, including those with KMT2A-r, indicating that HDAC8 inhibition could still restore p53 acetylation in this setting. Using both genetic deletion and pharmacological inhibition of HDAC8 in KMT2A::MLLT3-driven AML models, we demonstrate that HDAC8 is critical for leukemia progression. Notably, HDAC8 inhibition suppresses AML cell proliferation and survival across genetically diverse AML subtypes through downregulation of the STAT3–MYC axis, independent of p53 status. Furthermore, HDAC8 inhibition synergizes with Venetoclax in murine AML and PDX models, underscoring the translational potential of this combination strategy in AML.

HDAC8 overexpression has been reported in multiple malignancies, including breast cancer and neuroblastoma, where it correlates with drug resistance and poor prognosis [33, 34]. Although several pan-HDAC inhibitors have been approved for cancer treatment [35], these agents exhibit limited activity against HDAC8 and are frequently associated with dose-limiting toxicities [36]. Consequently, there is increasing interest in developing isoform-selective HDAC inhibitors to improve therapeutic specificity. Several selective HDAC8 inhibitors, including 22d [23] and PCI-34051 [25], have been developed based on the unique structural characteristics of HDAC8 [37, 38]. While restoration of p53 acetylation underlies the activity of HDAC8i in *TP53*-WT AML [14], our findings reveal additional p53-independent mechanisms that substantially broaden the therapeutic relevance of HDAC8 inhibition. Transcriptome analysis uncovered extensive transcriptional reprogramming following HDAC8 inhibition in *TP53*-null or *TP53*-MUT AML cells. Notably, induction of *CDKN1A* enforces a potent G_0_/G_1_ cell cycle arrest, while concurrent upregulation of the BH3-only family proteins BBC3, PMAIP1, BNIP3 and BNIP3L indicates activation of a p53-bypass intrinsic apoptotic program. Together, these mechanisms provide a molecular basis for the efficacy of HDAC8 inhibition in p53-aberrant AML.

Our transcriptomic and biochemical analyses identify STAT3 as a critical mediator linking HDAC8 activity to MYC-driven oncogenic programs. HDAC8 physically interacts with STAT3, suppressing STAT3 acetylation, and promotes STAT3 protein stability, thereby sustaining MYC expression and its downstream transcriptional network. Accordingly, inhibition of HDAC8 enhances STAT3 acetylation, accelerates STAT3 degradation and suppresses MYC-driven gene expression. Extensive dysregulation of MYC-activated and MYC-repressed genes was observed in both KMT2A-r and KMT2A-intact contexts (Fig. S4C, D), indicating that this mechanism operates independently of KMT2A status. Although the differentially expressed MYC target gene sets may be context-dependent, we observed a common set of differentially expressed genes (DEG) for both MYC-activated (e.g., *MYC*) and MYC-repressed (e.g., *CDKN1A)* genes (Table S10) regardless of KMT2A status. Importantly, this pathway is also *TP53-*independent, distinguishing it from the context-dependent p53 axis and positioning the STAT3–MYC pathway as a fundamental vulnerability in high-risk AML.

The HDAC8-regulated STAT3–MYC axis operates in parallel to the p53-dependent mechanism we previously reported [14, 17]. Together, these findings position HDAC8 as a central therapeutic node that simultaneously regulates two critical effector pathways in AML: the canonical p53 tumor-suppressor axis and the STAT3–MYC pro-survival axis. In *TP53*-WT AML, disruption of both pathways is likely to yield maximal therapeutic benefit. In *TP53*-MUT or *TP53*-null AML, where p53 signaling is compromised, leukemic cell survival remains critically dependent on STAT3–MYC signaling. This dual regulatory function provides a compelling explanation for the broad efficacy of HDAC8 inhibition across genetically heterogeneous AML subtypes.

Serial-transplantation experiments demonstrated that HDAC8 inhibition significantly prolonged survival of secondary transplant recipients in both murine AML and PDX models, indicating effective targeting of LSC function. Although only modest survival benefits were observed in a limited cohort of primary treated PDX mice (Fig. S14), optimization of treatment timing, dosage, and scheduling may enhance efficacy. Beyond its cell-intrinsic role in leukemic survival, STAT3 is a central regulator of the immunosuppressive tumor microenvironment in AML and other malignancies [39, 40]. Accordingly, inhibition of STAT3 signaling by HDAC8i may not only impair leukemic cell viability but also disrupt immune evasion mechanisms and potentiate anti-leukemic immune responses [41–43]. Consistent with our observation of modest B-cell reduction following HDAC8 inhibition or deletion [17], attenuation of STAT3 activity has been reported to preferentially affect mature B-cells while sparing hematopoietic progenitors [44, 45]. These findings highlight the HDAC8–STAT3 axis as a potential mediator of immune remodeling in AML, warranting further investigation.

Venetoclax has transformed the treatment landscape for AML with high initial response rates; however, its clinical benefit is often limited by the inevitable emergence of resistance driven by multiple molecular mechanisms [46–48]. Inactivation of the p53 apoptotic network can mediate Venetoclax resistance [46, 47, 49], while aberrant activation of STAT3 and MYC pathways promotes resistance through enhanced anti-apoptotic signaling and proliferative capacity [50, 51]. By suppressing STAT3–MYC signaling, inducing cell cycle arrest, and activating intrinsic apoptotic pathways, HDAC8 inhibition provides a strong mechanistic rationale for combination therapy with Venetoclax independent of *TP53* status. Notably, although HDAC8i-Venetoclax combination treatment modestly reduced lymphocyte numbers, particularly the circulating B-cells, this effect is consistent with the known immunologic profile of Venetoclax, and the addition of HDAC8i did not further compromise normal hematopoiesis.

The therapeutic promise of HDAC8 inhibition is further supported by prior studies showing its efficacy in inv(16) AML [14], in combination with TKI in FLT3-ITD^+^ AML [15] and in combination with nicotinamide phosphoribosyltransferase inhibition [52]. Together with our current findings, these data establish HDAC8 as a key, multifaceted regulator of leukemogenesis across diverse subtypes of AML. Importantly, we previously showed that *Hdac8* knockout mice exhibit normal long-term survival and largely intact hematopoiesis under homeostatic conditions [17], suggesting that on-target HDAC8 inhibition may be well-tolerated. Nevertheless, further studies are required to define predictive biomarkers of response, optimize therapeutic combinations, and evaluate long-term safety, drug interactions, and the impact on the tumor microenvironment [53–56].

In conclusion, our study identifies HDAC8 as a critical downstream effector of *KMT2A* fusion oncogenes that sustains leukemic propagation through the STAT3–MYC axis. These findings highlight HDAC8 inhibition as a promising therapeutic strategy, particularly in combination with Venetoclax, for high-risk AML and *TP53*-mutant AML.

## Supplementary information

Supplementary Information

Table S6

Table S7

Table S8

Table S9

Table S10

Supportive Data

## Data Availability

All data generated in this study are included in this article and its supplementary information. RNA-seq data have been deposited in the Gene Expression Omnibus (GEO) repository and are accessible with accession number GSE305655 (https://www.ncbi.nlm.nih.gov/geo/query/acc.cgi?acc=GSE305655).
