# Supplementary material for: HDAC8 inhibition targets STAT3–MYC axis and synergizes with Venetoclax in KMT2A-rearranged acute myeloid leukemia
Source: Leukemia. 2026 Apr 21;40(6):1271–83. doi: 10.1038/s41375-026-02950-1 (PMC13233295; doi:10.1038/s41375-026-02950-1)
Supplement: Supplementary file 1 — Supplementary Information [file 41375_2026_2950_MOESM1_ESM.pdf]

HDAC8 Inhibition Targets STAT3–MYC Axis and Synergizes with Venetoclax in KMT2A-Rearranged Acute Myeloid Leukemia

Lianjun Zhang[1#], Wancheng Guo[1#], Yu-Hsuan Fu[1,2#], Dijiong Wu[1,3#], Man Li[1], Ying-Chieh Chen[1], Chi-Yang Tseng[1], Wei-Kai Hua[1], Le Xuan Truong Nguyen[1], Xin He[1], Haojie Dong[1], Lei Zhang[1], Bin Zhang[1], Ling Li[1], Guido Marcucci[1] and Ya-Huei Kuo[1*]

Lists of Supplementary Information

Figures S1-14

Supplementary Methods

Table S1. Information for AML patient samples used

Table S2. List of antibodies used for flow cytometry

Table S3. The primer information for qPCR

Table S4. List of antibodies used for IP, IB and IF analysis

Table S5. The sequences for shRNAs

Table S6. RNA-seq data for MV4-11 cells treated with HDAC8i (22d) vs. vehicle

Table S7. RNA-seq data for KG-1 cells treated with HDAC8i (22d) vs. vehicle

Table S8. RNA-seq data for Kasumi-1 cells treated with HDAC8i (22d) vs. vehicle

Table S9. Hallmark pathways by gene set enrichment analysis (GSEA)

Table S10. HDAC8i (22d) induced differentially expressed genes (DEG) overlapping with MYC-activated or MYC-repressed targets

Reference

Supplementary Methods

Analysis of Public Dataset

Gene expression data of AML patients, presented as normalized transcript-per-million (TPM) were obtained from the TARGET (Therapeutically Applicable Research to Generate Effective Treatments) AML dataset (https://bioinformatics.mdanderson.org/MQA/)[1, 2] and clinical information was obtained from the Genomic Data Commons (GDC) portal (https://portal.gdc.cancer.gov/projects/TARGET-AML)[3-5]. Based on HDAC8 expression levels, patients with KMT2A-rearranged AML were classified into HDAC8-high and HDAC8-low groups using the MedianCutoff method. Survival analysis was performed using Kaplan-Meier analysis and Cox proportional hazards regression (R package survival v3.7-0; survminer v0.4.9). Gene expression data from GEO datasets (GSE131184, GSE114868, GSE76008, GSE68172, and GSE247175) were log2-transformed and normalized using the limma package, and Pearson correlation analysis was performed to investigate the relationship between SOX4 and HDAC8.

Bone Marrow Cell Isolation and Flow Cytometry

Mice were euthanized, and bone marrow was isolated after removing muscle and connective tissue. Bones were crushed using a mortar and pestle to obtain the single-cell suspension, which was subsequently treated with red blood cell lysis buffer to remove erythrocytes.

For fluorescence activated cell-sorting (FACS) analyses, cells were stained with

fluorescently labeled antibodies in phosphate-buffered saline (PBS) with 0.5% bovine serum albumin (BSA) for 15 minutes at 4 °C. Data were acquired using a 5-laser BD LSRFortessa™ X-20 flow cytometer (BD Biosciences, CA, USA).

For isolation of LSK (Lin^{-} Sca-1^{+} c-Kit^{+}) cells, lineage-negative (Lin^{-}) cells were stained with fluorochrome-conjugated streptavidin (Biolegend, San Diego, CA, USA), Sca-1, and c-kit antibodies (eBioscience, San Diego, CA, USA) and sorted using FACSAria™ Fusion cell sorter (BD Biosciences, CA, USA). Lineage-positive (Lin+) cell depletion was performed by incubating bone marrow cells with a cocktail of biotinylated monoclonal antibodies against CD19, NK1.1, Ter-119, B220, IgM, CD3, CD11b, Gr1, CD127, CD11c, CD41 (Biolegend, San Diego, CA, USA), followed by removal using the EasySep™ Mouse Streptavidin RapidSpheres™ kit (STEMCELL Technologies, Vancouver, BC, Canada). Antibodies used for flow cytometry are listed in Supplementary Table 2.

Generation of *KMT2A*-rearranged Murine Model

MSCV-based MIG-HA-Bio, IRES-GFP-*KMT2A::MLLT3*-HA-Bio, and *KMT2A::MLLT1*-HA-Bio vectors were used to generate KMT2A-rearranged murine cells by retroviral transduction of LSK cells. Retrovirus was produced by co-transfecting Phoenix cells with the pCL-Eco packaging plasmid using polyethylenimine (PEI, Millipore Sigma, Burlington, MA). Virus-containing supernatants were collected 2-3 days post-transfection, and viral particles were concentrated using Retro-X Concentrator (TaKaRa Bio, Kusatsu, Japan).

LSK cells were isolated from bone marrow of wild-type mice and cultured overnight in IMDM medium supplemented with 1% penicillin/streptomycin (P/S), 10% fetal bovine serum (FBS) with cytokines including murine Interleukin-3 (IL-3, 10 ng/mL), Interleukin-6 (IL-6, 6 ng/mL), Stem Cell Factor (SCF, 20 ng/mL), and Thrombopoietin (TPO, 20 ng/mL). For retroviral transduction, non-tissue culture-treated 24-well plates were first coated with Retronectin (12.5 μg/mL, Takara Bio, Japan) at 4°C overnight, then blocked with 2% BSA in PBS for 30 minutes at room temperature. High-titer retrovirus was loaded onto the Retronectin-coated wells by centrifugation at 1500 × g for 90 minutes at 32°C (Beckman Coulter, CA, USA). LSK cells were resuspended at a density of 0.5 × 10^6 cells/mL and added to the virus-coated wells at a multiplicity of infection (MOI) of 10. Plates were centrifuged again at 1500 × g for 90 minutes at 32°C and incubated at 37°C with 5% CO_2. After 48 hours of transduction, GFP^+ cells were sorted and either analyzed directly to assess *Hdac8* and *Sox4* expression or transplanted into sublethal-irradiated (4.5 Gy) C57BL/6 wild-type recipient mice via tail vein injection (2×10^5 GFP^+ cells/mouse) for leukemia induction.

KMT2A::MLLT3 Murine Leukemia Model with Inducible _Hdac8_ Deletion

To generate inducible *Hdac8* deletion in the *KMT2A::MLLT3* murine model, LSK cells were isolated from a conditional *Hdac8*-deletion [*Mx1-Cre/Hdac8*$^{f/f(y)}$] mice and transduced with MSCV-*KMT2A::MLLT3* retroviral vector[6]. Two days after transduction, GFP^+ cells were sorted and transplanted, together with supporting cells, into lethally irradiated (4.5 Gy + 4.5 Gy) C57BL/6 wild-type recipients via tail vein injection. Upon

disease progression, *Hdac8* deletion was induced in leukemic cells derived from *Mx1-Cre/Hdac8*$^{f/f(y)}$ donors by intraperitoneal (i.p.) injection of poly(I:C) at 14 mg/kg/dose every day for two weeks. Transplanted mice were monitored by assessing peripheral blood white blood cell counts, frequency of GFP^+ leukemia cells, and overall survival.

Virus Packaging and Cell Transduction

The packaging plasmids (psPAX2 and pMD2.G) were obtained from Addgene (Cambridge, MA, USA) and *Sox4* and *STAT3* shRNA vectors (sequence listed in supplementary Table S5), as well as the corresponding scramble control plasmids were purchased from Millipore Sigma (Burlington, MA). The HDAC8 overexpression (OE) plasmid was constructed using the CD531A-2 (pCDH-EF1α-MCS-IRES-RFP) backbone. The HDAC8 coding sequence (CDS) was inserted into the vector through the XbaI and EcoRI restriction sites to generate the pCDH-EF1α-HDAC8-IRES-RFP plasmid. All plasmids were transformed and amplified in Stbl3 competent cells.

For lentiviral production, transgene, psPAX2, and pMD2.G plasmids were co-transfected into adherent 293T cells (ATCC, Manassas, VA, USA) at a 3:2:1 ratio using polyethylenimine. After transfection (8 hours), the culture medium was replaced, and viral supernatants were collected every 24 hours for three consecutive days. The viral supernatant was centrifuged at 1000 × g for 15 minutes to remove cell debris.

For *Sox4* knockdown, 32D murine myeloid progenitor cells were transduced using Retronectin-based method (TaKaRa Bio, Kusatsu, Japan) above described following the manufacturer's instructions. For *STAT3* knockdown, MV4-11 cells were infected

with the lentiviral supernatant using polybrene (Sigma-Aldrich) to enhance transduction efficiency. Cells were spin-infected at 1500 × g for 2 hours, with medium changed after 24 hours. Cells were then cultured for an additional 12 hours prior to selection with 1 μg/mL puromycin, which was maintained for 48 hours. Knockdown efficiency was verified by quantitative PCR (qPCR).

Dual-Luciferase Reporter Assay

To investigate whether SOX4 directly regulates HDAC8 transcription, the *HDAC8* promoter region (-2000 bp to +193 bp relative to the transcription start site) was amplified using the following primers: forward 5'-TTTTACGCGTCTAAGCATCCTACATTCATT-3' (MluI site) and reverse 5'-TTTTGCTCGAGCCGTTTGGGGATCTTGGCCA-3' (HindIII site). The PCR product was cloned into the pGL3-basic vector (Promega, Madison, WI, USA) to generate the HDAC8 luciferase reporter construct (HDAC8-pGL3). Putative SOX4 binding sites within this promoter region were identified using the JASPAR database.

HEK293T cells were co-transfected with the HDAC8-pGL3 reporter plasmid and the SOX4 overexpression plasmid (SOX4-pcDNA3.1) or empty vector control using Lipofectamine™ 2000 Transfection Reagent (Thermo Fisher Scientific, CA, USA) according to the manufacturer's instructions. Cells were harvested 48 hours post-transfection and luciferase activity was measured using the Dual-Luciferase Reporter Assay System (Promega, Madison, WI, USA) according to the manufacturer's instructions. Firefly luciferase activity was normalized to Renilla luciferase activity to

control for transfection efficiency.

Chromatin Immunoprecipitation (ChIP)

Human HEK293T cells expressing SOX4–FLAG were collected and subjected to ChIP assays. HEK293T cells were cultured in DMEM supplemented with 10% fetal bovine serum (FBS) and transfected with SOX4-FLAG in the pcDNA3.1 vector (Addgene, Cambridge, MA, USA) using polyethylenimine at a 3:1 PEI:DNA ratio. After 8 hours of transfection, the medium was replaced, and cells were harvested 48 hours post-transfection.

ChIP was performed using the SimpleChIP® Plus Chromatin Immunoprecipitation Kit (Magnetic Beads) following the manufacturer's instructions (Cell Signaling Technology, Danvers, MA, USA). Cells were cross-linked with 1% formaldehyde for 10 minutes at room temperature, quenched with glycine. Chromatin was enzymatically digested to an average fragment size of 150–900 bp. ChIP was performed using an anti-FLAG antibody (Cell Signaling Technology, MA), and normal rabbit IgG was used as a negative control. After de-crosslinking and DNA purification, quantitative PCR (qPCR) was performed using SYBR Green Master Mix with primers targeting two different HDAC8 promoter regions (Supplementary Table S3). The enrichment of DNA was calculated using the percent input method:

$\% \text{ Input} = 2\% \times 2^{(\text{Ct Input} - \text{Ct IP})}$.

Flow Cytometry Analysis for Apoptosis

Apoptosis was assessed using flow cytometry with the Annexin V/DAPI staining. Annexin V+ / DAPI− cells were classified as early apoptotic, and Annexin V+ / DAPI+ cells were considered late apoptotic. Flow cytometry analysis was performed using the BD LSRFortessa X-20 flow cytometer (BD Biosciences, CA, USA).

Colony Formation Assay

KMT2A::MLLT3 leukemic mouse bone marrow cells were treated with vehicle (DMSO) or the HDAC8 inhibitor 22d (10 μM) for 48 h. Cells were then plated in MethoCult™ methylcellulose medium (M3534; STEMCELL Technologies, Canada) at a density of 200 viable cells/mL in 3.5-cm dishes. Colonies were counted on day 7.

Cell Culture and Drug Treatment

Human leukemia cell lines, including Molm-13, MV4-11, NB4, Kasumi-1, THP-1, KG-1, and MONO-MAC6, were obtained from the American Type Culture Collection (ATCC, Manassas, VA, USA) and cultured in RPMI 1640 medium (Corning, NY, USA) supplemented with 10% fetal bovine serum. Primary AML cells were cultured in StemSpan™ medium (STEMCELL Technologies, Canada) supplemented with the following cytokines: GM-CSF (200 pg/mL, granulocyte-macrophage colony-stimulating factor), G-CSF (1ng/mL, granulocyte colony-stimulating factor), SCF (200 pg/mL), IL-6 (1 ng/mL), MIP-1α (200 pg/mL, macrophage inflammatory protein-1α), and LIF (50 pg/ml, leukemia inhibitor factor). Clinical information was summarized in Supplementary table 1. All the cultures were maintained in a 37°C, 5% CO_2 incubator.

For drug treatments, cells were exposed to HDAC8 inhibitor 22d (MedKoo Biosciences, NC) and/or the BCL2 inhibitor Venetoclax (LC Labs, MA) in the culture medium for indicated time and concentrations. DMSO (Thermo Fisher Scientific, MA) was used as vehicle control.

Cell Viability Assay

Cell viability was assessed by an ATP assay using the CellTiter-Glo® Luminescent Cell Viability Assay Kit (Promega, WI) following manufacture's protocol. In brief, CellTiter-Glo reagent was added to the culture wells at a 1:1 volume ratio and incubated for 10 minutes at room temperature to ensure complete cell lysis and substrate conversion. Relative viability was calculated by normalizing luminescence to untreated controls. All measurements were done in triplicate and analyzed with Gen5 software (Molecular Devices, CA).

Cell Cycle Analysis with EdU and Propidium Iodide (PI) Staining

Cell proliferation and cell cycle distribution were assessed using the Click-iT™ EdU Alexa Fluor™ 405 Flow Cytometry Assay Kit (Thermo Fisher Scientific, USA) in combination with PI staining. Suspension cells were incubated with 10 µM EdU for 2 hours at 37 °C in a 5% CO_2 incubator. After labeling, cells were collected by centrifugation at 300 × g for 5 minutes and washed once with PBS. Cells were fixed in 4% paraformaldehyde for 15 minutes at room temperature, then permeabilized with 0.5% Triton™ X-100 in PBS for 20 minutes. The Click-iT™ reaction cocktail was

prepared according to the manufacturer's instructions and added to the cell pellet. Cells were incubated in the dark for 30 minutes at room temperature. After the Click-iT™ reaction, cells were washed once with PBS, then incubated with 50 μg/mL PI and 100 μg/mL RNase A for 30 minutes at room temperature in the dark for DNA content analysis. Flow cytometry was performed using a BD LSRFortessa X-20 (BD Biosciences, CA) and analyzed with FlowJo (v10.8.1, BD Biosciences, CA).

Quantitative RT-PCR

Total RNA was isolated using Trizol reagent (Invitrogen, MA, USA) with the Direct-zol RNA Microprep kit (ZYMO, CA) following the manufacturer's protocol. First-strand cDNA synthesis was performed with SuperScript IV reverse transcriptase (Thermo Fisher Scientific, USA). Quantitative RT-PCR was performed on the QuantStudio 7 Flex Real-Time PCR System (Thermo Fisher Scientific, USA) using the SYBR Green I master mix (Thermo Fisher Scientific, USA) or TaqMan™ Universal Master Mix II, with UNG (Thermo Fisher Scientific, USA), according to the manufacturer's instructions. *ACTB* or *B2m* was used as internal control. Relative gene expression levels were calculated using the Ct method ($2^{-}\Delta Ct$) after normalization to the internal control. Primer information was listed in Supplementary Table 3.

Western Blotting

Total protein was first extracted from the cells, and protein concentration was determined using the BCA assay. Protein samples were separated by electrophoresis

using 10% SDS-PAGE gel (Bio-rad, CA, USA), followed by transfer to PVDF membranes (Bio-rad, CA). The transferred membranes were blocked for 1 hour with 5% non-fat milk in PBST buffer to block non-specific binding. The membranes were then incubated overnight at 4°C with primary antibodies, diluted according to the manufacturer's instructions. The following day, the membranes were washed three times using PBST, (10 minutes each), and subsequently incubated with HRP-conjugated secondary antibodies at room temperature for 1 hour, followed by three additional washes (15 minutes each). Finally, ECL (Thermo Fisher Scientific, USA) or WestFemto (Thermo Fisher Scientific, USA) chemiluminescence detection reagents were applied, and detected signal was captured using GeneSys Imaging System (GeneSys, UK). The grayscale intensity of protein bands was quantified using the ImageJ software (NIH, USA) for relative densitometric analysis. Antibodies used are listed in Supplementary Table 4.

Cycloheximide (CHX) Chase assay for STAT3

To examine the impact of HDAC8i (22d) on STAT3 protein stability, MONO-MAC6 cells were divided into four groups and treated with vehicle control; HDAC8i (22d, 10 μM); CHX (100 μg/Ml, Sigma); and the combination of HDAC8i (22d, 10 μM) and CHX (100 μg/mL). Cells were harvested at 0, 4, 6, 8, 12, and 24 hours after treatment for protein extraction and subjected to immunoblotting analysis of STAT3 or HSP90 as internal control.

Co-Immunoprecipitation (Co-IP) Analysis

Co-Immunoprecipitation (Co-IP) was employed to analyze HDAC8 and STAT3 protein-protein interaction. Cell lysates were prepared by sonication using a lysis buffer containing protease inhibitors (Thermo Fisher Scientific, USA; diluted 1:100) then incubate with indicated antibodies for protein pulldown, including HDAC8 antibodies (Abcam, MA), or STAT3 antibodies (Cell Signaling Technology, MA) with rotation in 4° for overnight, followed by the incubation with protein A/G agarose beads (Millipore Sigma, Burlington, MA) for 2 hours. After incubation, the protein-beads complexes were washed multiple times with a low-salt wash buffer to remove non-specifically bound proteins. Finally, the proteins were eluted using a protein sample buffer and analyzed by SDS-PAGE. Similarly, to access the acetylation status of STAT3, acetylation-specific antibodies (Abcam, MA) were used for immunoprecipitation, followed by probing with STAT3 antibodies (Cell Signaling Technology, MA).

RNA Sequencing and Data Analysis

MV4-11, KASUMI-1, and KG-1 cells were treated with 10 μM HDAC8i (22d) or DMSO for 12 hours. Following treatment, total RNA was extracted from the cells using the Direct-zol RNA Miniprep-Kit (Zymo research, CA), and RNA sequencing libraries were constructed using the KAPA RNA HyperPrep (Roche, Switzerland) with RiboErase. The libraries were then sequenced on the Illumina NovaSeq 6000 platform with paired-end sequencing. Raw sequencing data were processed using Trimmomatic and FASTP for trimming, removing adapters and polyA tails. The cleaned

data were then aligned to the human genome (hg38) using the STAR software. Subsequently, low expressing genes were filtered out with filterByExpr function (with minmin.count = 1 and min.prop = 0.7) in the edgeR package (v4.8.2), and the filtered count data were subsequently normalized using the normalizeBetweenArrays function from the limma package (v3.66.0). Differential expression analysis was performed with the eBayes method in limma, and genes with an adjusted p-value (FDR) < 0.05 were considered significantly differentially expressed genes (DEG). Gene set enrichment analysis (GSEA) was conducted by clusterprofiler (v4.18.4) based on the log2 fold change of genes, using pre-ranked analysis to examine the enrichment of Hallmark pathways (from the msigdbr package, v25.1.1). The hit ratio for each gene set was defined as the proportion of leading-edge genes in the gene set. HALLMARK MYC TARGETS V1 and V2 gene sets (M5926 and M5928) were considered MYC-activated genes and intersected with down-regulated DEG (DEG-down) from each cell line; DANG MYC TARGETS DN gene set (M2310) were considered MYC-repressed genes and intersected with up-regulated DEG (DEG-up) from each cell line. The DEG-down and DEG-up gene lists for each cell line were shown in Table S10. All RNA sequence data were uploaded to GSE305655.

KMT2A::MLLT3 Murine Leukemia Model

To establish the KMT2A::MLLT3 leukemia cohort, leukemic bone marrow cells were collected from primary leukemic mice and transplanted into non-irradiated C57BL/6 wild-type recipient mice. Seven days after transplantation, when the

frequency of GFP$^+$ leukemic cells in peripheral blood exceeded 5%, mice were randomly assigned to treatment groups, including vehicle control, HDAC8 inhibitor (22d, 50 mg/kg, i.p., BID), Venetoclax (100 mg/kg, oral gavage, QD) or combination therapy (same dose/schedule as single agent) and treated for two weeks. Following treatment, some of the mice were monitored for survival, while the remaining mice were euthanized and BM was harvested for flow cytometry analysis and secondary transplantation. For secondary transplantation, BM cells (2 x 10^6 cells) from each treatment group were transplanted into C57BL/6 wild-type mice, which were monitored for leukemia recurrence and survival (time to moribund endpoint).

AML Patient-Derived Xenograft (PDX) Model

To establish patient-derived AML models, 6–8 weeks irradiated (2 Gy) NOD/SCID/IL-2R-$\gamma^{-/-}$/Tg (CMV-IL3, CSF2, KITLG) (NSGS; Jackson Laboratory, #013062) mice were intravenously (i.v.) transplanted with T cell-depleted AML cells (2 x 10^6). Primary AML cells were depleted of T cells using magnetic-activated cell sorting (Miltenyi Biotec, Germany) with CD3 MicroBeads (Miltenyi Biotec, Germany). When the proportion of human CD45$^+$ AML cells in peripheral blood approached ~3%, mice were randomly assigned to treatment groups as described above. Following treatment, some of the mice were monitored for survival, while the remaining mice were euthanized for assessment of leukemia burden and secondary transplantation. Leukemia burden was evaluated by staining with human CD34, CD33, and CD45 antibodies (BD Biosciences, CA) followed by flow cytometry. For secondary

transplantation, BM cells (2 x 10^6 cells) from each treated group were transplanted into irradiated NSGS recipients as described above. Leukemia burden in secondary recipients was assessed by flow cytometry of peripheral blood over time. For survival analysis, mice were observed daily and the time to moribund endpoint was recorded.

Toxicity Assessment

For toxicity evaluation, wild-type mice were treated with the same dose/schedule of drugs for two weeks as described above. Blood samples were collected every two weeks for complete blood count (CBC) analysis and flow cytometric assessment. Ten weeks after treatment, mice were euthanized, and BM was harvested for analysis of hematopoietic stem and progenitor cell and stromal cell populations. Flow cytometry data were acquired using the BD Fortessa X-20 flow cytometer (BD Biosciences, CA). Information of antibodies used for flow cytometry is listed in Table S2.

Reference

1. Brunner AM, Graubert TA. Genomics in childhood acute myeloid leukemia comes of age. *Nat Med* 2018 Jan 9; **24**(1)**:** 7-9.

2. Bolouri H, Farrar JE, Triche T, Jr., Ries RE, Lim EL, Alonzo TA*, et al.* The molecular landscape of pediatric acute myeloid leukemia reveals recurrent structural alterations and age-specific mutational interactions. *Nat Med* 2018 Jan; **24**(1)**:** 103-112.

3. Tyner JW, Tognon CE, Bottomly D, Wilmot B, Kurtz SE, Savage SL*, et al.* Functional genomic landscape of acute myeloid leukaemia. *Nature* 2018 Oct; **562**(7728)**:** 526-531.

4. Bolouri H, Farrar JE, Triche T, Jr., Ries RE, Lim EL, Alonzo TA*, et al.* The molecular landscape of pediatric acute myeloid leukemia reveals recurrent structural alterations

and age-specific mutational interactions. *Nat Med* 2018 Jan; **24**(1)**:** 103-112.

5. Bottomly D, Long N, Schultz AR, Kurtz SE, Tognon CE, Johnson K*, et al.* Integrative analysis of drug response and clinical outcome in acute myeloid leukemia. *Cancer Cell* 2022 Aug 8; **40**(8)**:** 850-864 e859.

6. Krivtsov AV, Twomey D, Feng Z, Stubbs MC, Wang Y, Faber J*, et al.* Transformation from committed progenitor to leukaemia stem cell initiated by MLL-AF9. *Nature* 2006 Aug 17; **442**(7104)**:** 818-822.

Table S1. Information for AML patient samples used

Sample ID	Sex	Sample Type	Disease Status	P53 Status	Cytogenetic/Mutation
AML 1180	F	PB	New Diagnosis	WT	Normal
AML 1953	F	PB	AML	MUT	Abnormal female karyotype. Positive for monosomy 5, TP53 deletion, and KMT2A (MLL) gain and amplification by interphase FISH analyses
AML 2388	M	PB	AML	WT	Normal male karyotype. Positive for trisomy 9 and tetrasomy 22, and KMT2A (MLL) gain by interphase FISH analyses
AML 1920	M	PB	AML	MUT	MLL deletion detected by FISH analysis [95.0%]
AML 709	F	PB	AML	WT	Normal karyotype
AML 0276	F	PB	AML	WT	Stemline: 46,XX,t(9;11)(p22;q23 [5]); Sideline: 46,sl,t(1;4)(p36.1;q21 [2]); Nonclonal: t(5;17)(q11.2;p13); KMT2A FISH: 12.4% translocation
AML 741	M	PB	AML	WT	Trisomy 11 in 13/20 cells; MLL gain [37.1%] by FISH
AML 1807	F	PB	AML	WT	FLT-3 ITD Neg., FLT-3 D835 Neg., NPM1 Neg.
AML 0328	M	PB	Refractory	MUT	Complex karyotype with del(5q), del(7q), TP53 loss, RUNX1T1 gain, RARA rearrangement neg
AML 1298	M	PB	Relapsed	Unknow	t(16;16), Trisomy 22; FLT-3 ITD Neg., FLT-3 D835 Pos., NPM1 Neg., C-kit Neg
AML 357	M	PB	New Diagnosis	Unknow	45, XY, t(3;8) (q26 .2; q24.2), -7[20]; PTPN11 (c.205G>A; p.E69K) 33%; NRAS (c.182A>T; p.Q61L) 3%
AML 987	M	PB	Relapsed	Unknow	t(16;16), Trisomy 22; FLT-3 ITD Neg., FLT-3 D835 Pos., NPM1 Neg., C-KIT Neg.
AML 1160		PB	Relapsed	Unknow	Monosomy7
AML 1147	F	PB	Refractory	Unknow	t(8;21); FLT-3 ITD Pos. FLT-3 D835 Neg.
AML 496	M	PB	Relapsed	MUT	Clone 1: 46,XY,t(12;13)(p12.2;q12.3)[13] Clone 2: 46,XY,t(2;12)(q33p13.2)[2] Constitutional cell line: 46,XY[8] ZRSR2 (c.1207del; p.R403Gfs*?) 27%; MPL (c.1543T>A; p.W515R) 6%; DNMT3A (c.1015-1G>A) 4%; FLT3 (c.1794_1795ins36; p.E598_Y599ins12) 3%
AML 075	M	BM	Relapsed	MUT	46,XY,del(17)(p11.2)[16] Positive for loss of TP53; TP53 (c.701A>G; p.Y234C)

					52%; U2AF1 (c.467G>A; p.R156H) 47%; ASXL1 (c.2728C>T; p.Q910*) 47%; RUNX1 (c.593A>G; p.D198G) 30%; FLT3 (c.1837+1_1837+2ins57) N/A<br><br>FLT3 INTERNAL TANDEM DUPLICATION (ITD) CONFIRMED BY PCR WITH A SIGNAL RATIO OF 16.5
AML 773	M	PB	Relapsed	WT	Normal karyotype; BRCA2 mutation
AML 016	F	PB	New Diagnosis	WT	Normal karyotype; DNMT3A (c.2207G>A; p.R736H) 38%; FLT3 (c.2508_2510del; p.I836del) 20%; NPM1 (c.860_863dupTCTG; p.W288Cfs*12) 5%
AML 0618	F	PB	Relapsed/ Refractory	MUT	Complex karyotype; FISH: del(5q) (87%), del(7q) (88%), KMT2A gain/tetrasomy/amplification, trisomy 8, TP53 loss (91%)
AML 0506	F	PB	Newly diagnosed	MUT	46,XX[20]; FISH neg: MECOM, del(5q), -7, KMT2A gain, del(20q12)

Table S2. List of antibodies used for flow cytometry

Antibody name	Information
Biotin anti-mouse CD19, Clone: 6D5	1:400, (BioLegend Cat# 115504, RRID: AB_313639)
Biotin anti-mouse NK1.1, Clone: PK136	1:400, (BioLegend Cat# 108704, RRID: AB_313391)
Biotin anti-mouse TER-119, Clone: TER-119	1:200, (BioLegend Cat# 116204, RRID: AB_313705)
Biotin anti-mouse/human CD45R/B220, Clone: RA3-6B2	1:200, (BioLegend Cat# 103204, RRID: AB_312989)
Biotin anti-mouse IgM, Clone: RMM-1	1:200, (BioLegend Cat# 406504, RRID: AB_315054)
Biotin anti-mouse CD3, Clone: 17A2	1:50, (BioLegend Cat# 100244, RRID: AB_2563947)
Biotin anti-mouse/human CD11b, Clone: M1/70	1:100, (BioLegend Cat# 101204, RRID: AB_312787)
Biotin anti-mouse Ly-6G/Ly-6C (Gr-1), Clone: RB6-8C5	1:100, (BioLegend Cat# 108404, RRID: AB_313369)
APC-eFluor 780 anti-mouse CD117 (c-Kit), Clone: ACK2	1:100, (Thermo Fisher Scientific Cat# 47-1172-82, RRID: AB_1582226)
Alexa Fluor 488 anti-mouse Ly-6A/E (Sca1), Clone: E13-161.7	1:100, (BioLegend Cat# 122516, RRID: AB_756201)
Brilliant Violet 605 Streptavidin	1:100, (BioLegend Cat# 405229)
Brilliant Violet 605 anti-human CD33, Clone: P67.6	1:100, (BioLegend Cat# 366612, RRID: AB_2566405)
PE-CY7 anti-human CD34, Clone: 4H11	1:100 (Thermo Fisher Scientific Cat# 25-0349-42, RRID:AB_1963576)
APC anti-human CD45, Clone: 2D1	1:100, (BioLegend Cat# 368512, RRID: AB_2566372)
PE Annexin V	5 μL/test, (BioLegend Cat# 640947)
DAPI	1 μg/mL, (Thermo Fisher Cat# P36941)

Table S3. The primer information for qPCR

**Name**	**Sequence**
Human	
HDAC8 F	GGTGACGTGTCTGATGTTGG
HDAC8 R	GACACTTGCCAATTCCCACT
ACTIN F	GTGGATCAGCAAGCAGGAG
ACTIN R	TTTGTCAAGAAAGGGTGTAACG
MYC F	CATCAGCACAACTACGCAGC
MYC R	GCTGGTGCATTTTCGGTTGT
NPM1 F	GTTCTCTGGAGCAGCGTTCT
NPM1 R	TTTGCACCAGCCCCTAAACT
CDKN1a (p21)	TACCCTTGTGCCTCGCTCAG
CDKN1a (p21)	CGGCGTTTGGAGTGGTAGA
BNIP3 F	CAGGGCTCCTGGGTAGAACT
BNIP3 R	CTACTCCGTCCAGACTCATGC
BNIP3L F	CTGAGTGCCGGAGACGGTCC
BNIP3L R	CTGCCATCTTCTTGTGGCGAAGG
PUMA F	GACCTCAACGCACAGTACGAG
PUMA R	AGGAGTCCCATGATGAGATTGT
Murine TaqMan	
Sox4	Mm00486320_s1
Hdac8	Mm01224980_m1
Myc	Mm00487804_m1
Npm1	Mm02391781_g1
E2f1	Mm00432939_m1
Cdk4	Mm00726334_s1
Bax	Mm00432051_m1
Bbc3	Mm00519268_m1
Pmaip1	Mm00451763_m1
Bak1	Mm00432045_m1
B2m	Mm00437762_m1
ChIP primers	
HDAC8-A1 F (-171 to -68)	TTCATCTCAAATGGCCCCGTA
HDAC8-A1 R (-171 to -68)	CAAATAGCTGTCAATTAGGA
HDAC8-A2 F (-1399 to -1299)	AGGGATACAAAATGGAGGGT
HDAC8-A2 R (-1399 to -1299)	CACTGAAGCCTTTGAACATT

Table S4. List of antibodies used for IP, IB and IF analysis

No	Antibody name	Information
1	HSP90 alpha/beta Antibody (F-8)	1:1000 / IB; Santa Cruz Biotechnology Cat# sc-13119, RRID:AB_675659
2	β-Actin (13E5) Rabbit mAb	1:2000 / IB; Cell Signaling Technology Cat# 4970, RRID:AB_2223172
3	STAT3 (124H6) Mouse mAb	1:1000 / IB; 2 μg / IP; Cell Signaling Technology Cat# 9139, RRID:AB_331757
4	Anti-c-Myc antibody [Y69]	1:1000 / IB; Abcam Cat# ab32072, RRID:AB_731658
5	p53 Antibody (FL-393)	1:1000 / IB; Santa Cruz Biotechnology Cat# sc-6243, RRID:AB_653753
6	Acetyl-p53 (Lys382) Antibody	1:1000 / IB; Cell Signaling Technology Cat# 2525, RRID:AB_330083
7	Acetylated-Lysine (Ac-K2-100) MultiMab® Rabbit mAb mix	1:1000 / IB, 2 μg / IP; Cell Signaling Technology Cat# 9814, RRID:AB_10544700
8	HDAC8 Antibody	1:5000 / IB, 2 μg / IP; Abcam Cat# Ab187139, RRID:AB_2715505
9	CDK4 (D9G3E) Rabbit mAb	1:1000 / IB; Cell Signaling Technology Cat# 12790, RRID:AB_2631166
10	CDK6 (D4S8S) Rabbit mAb	1:1000 / IB; Cell Signaling Technology Cat# 13331, RRID:AB_2721897
11	Cyclin D3 (DCS22) Mouse mAb	1:1000 / IB; Cell Signaling Technology Cat# 2936, RRID:AB_2070801
12	Cleaved Caspase-3 (Asp175) Antibody	1:1000 / IB; Cell Signaling Technology Cat# 9661, RRID:AB_2341188
13	DYKDDDDK Tag (clone D6W5B) Rabbit monoclonal antibody	2 μg/ChIP; Cell Signaling Technology Cat# 14793, RRID:AB_2572291

Table S5. The sequences for shRNA

	Sequence
shSox4-TRCN0000012078	5'-CCGG-CAAGAAAGTGAAGCACGTCTA-CTCGAG-TAGACGTGCTTCACTTTCTTG-TTTTT-3'
shSox4-TRCN0000218406	5'-CCGG-TAAAGACCGAAGGAATCTTTC-CTCGAG-GAAAGATTCCTTCGGTCTTTA-TTTTTTGAAT-3'
shSTAT3-TRCN0000329887	5'-CCGG-GCACAATCTACGAAGAATCAA-CTCGAG-TTGATTCTTCGTAGATTGTGC-TTTTTG-3'
shSTAT3-TRCN0000020843	5'-CCGG-GCAAAGAATCACATGCCACTT-CTCGAG-AAGTGGCATGTGATTCTTTGC-TTTTT-3'

Figure S1

A

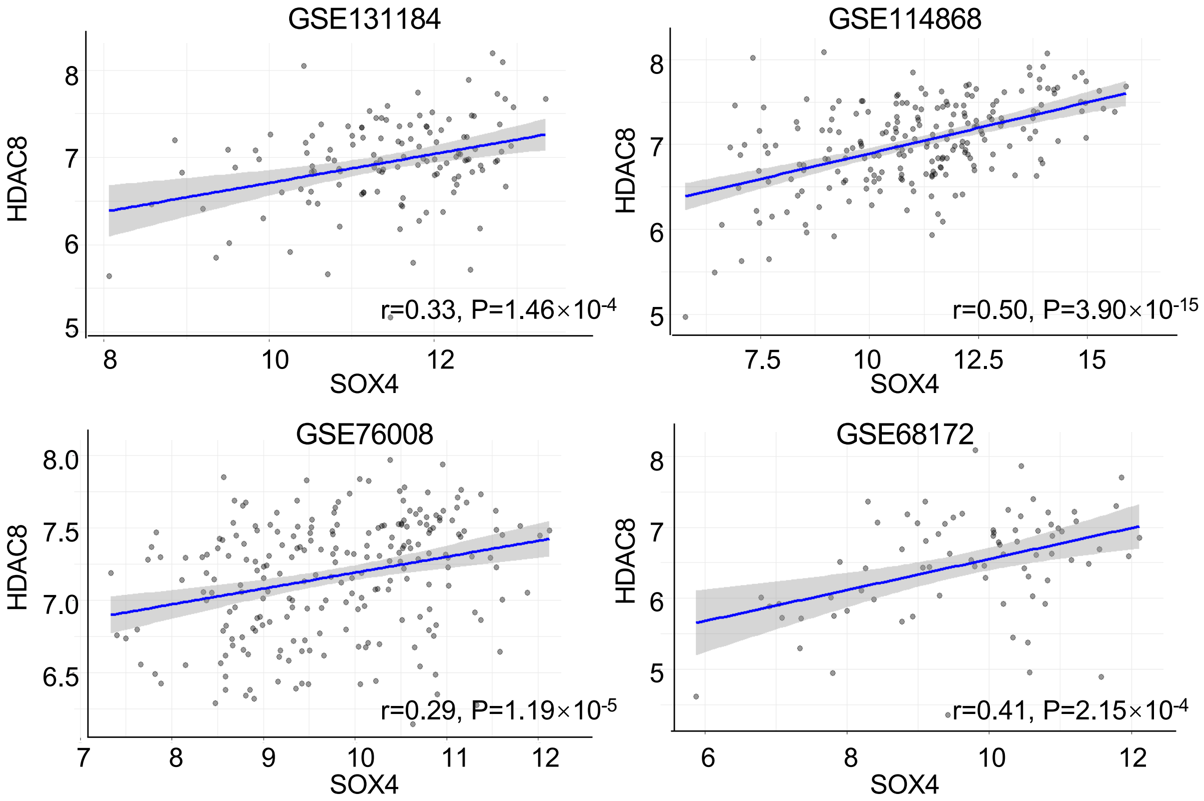

B

Matrix ID	Name	Score	Relative score	Sequence ID	Start	End	Strand	Predicted sequence
MA0867.3	MA0867.3.SOX4	9.094124	0.9205744	seq1	622	629	+	aacaaagt
MA0867.2	MA0867.2.SOX4	8.392172	0.8908677	seq1	621	630	+	gaacaaagtt
MA0867.3	MA0867.3.SOX4	6.56635	0.8724669	seq1	640	647	+	cacaaagt
MA0867.3	MA0867.3.SOX4	6.557332	0.8722953	seq1	673	680	+	aataaagg
MA0867.3	MA0867.3.SOX4	6.2237306	0.8659464	seq1	1758	1765	+	aacaagga
MA0867.3	MA0867.3.SOX4	6.184502	0.8651998	seq1	1801	1808	+	aacaatcg
MA0867.2	MA0867.2.SOX4	6.603803	0.858864	seq1	639	648	+	acacaaagtc
MA0867.2	MA0867.2.SOX4	6.52823	0.8575116	seq1	1800	1809	+	aaacaatcga
MA0867.2	MA0867.2.SOX4	6.5199213	0.8573629	seq1	672	681	+	taataaaggg
MA0867.2	MA0867.2.SOX4	6.2155004	0.8519152	seq1	1408	1417	-	gcacaaatgg

Figure S1. Correlation analysis of HDAC8 and SOX4 in AML samples and predicted SOX4 binding sites within *HDAC8* promotor.

A. Scatter plot showing the expression values of SOX4 (x-axis) and HDAC8 (y-axis) across four AML GEO datasets (GSE131184, GSE114868, GSE76008, GSE68172). Each dot represents one sample. The line indicates the linear regression fit.

B. Predicted SOX4 transcription factor binding sites within the *HDAC8* promoter region identified using JASPAR database. The table includes matrix IDs, transcription factor names, binding scores, relative scores, start and end positions on the promoter, and the corresponding predicted DNA sequences.

Figure S2

A

Cell lines	*TP53* status	TP53 function
MV4-11, Molm-13	Heterozygous P72R mutation	TP53 WT
MONO-MAC6	Heterozygous R273H	TP53 MUT
Kasumi-1, NB4	Homogeneous R248Q mutation	TP53 MUT
THP-1, KG-1	Absence of P53 protein	TP53 null

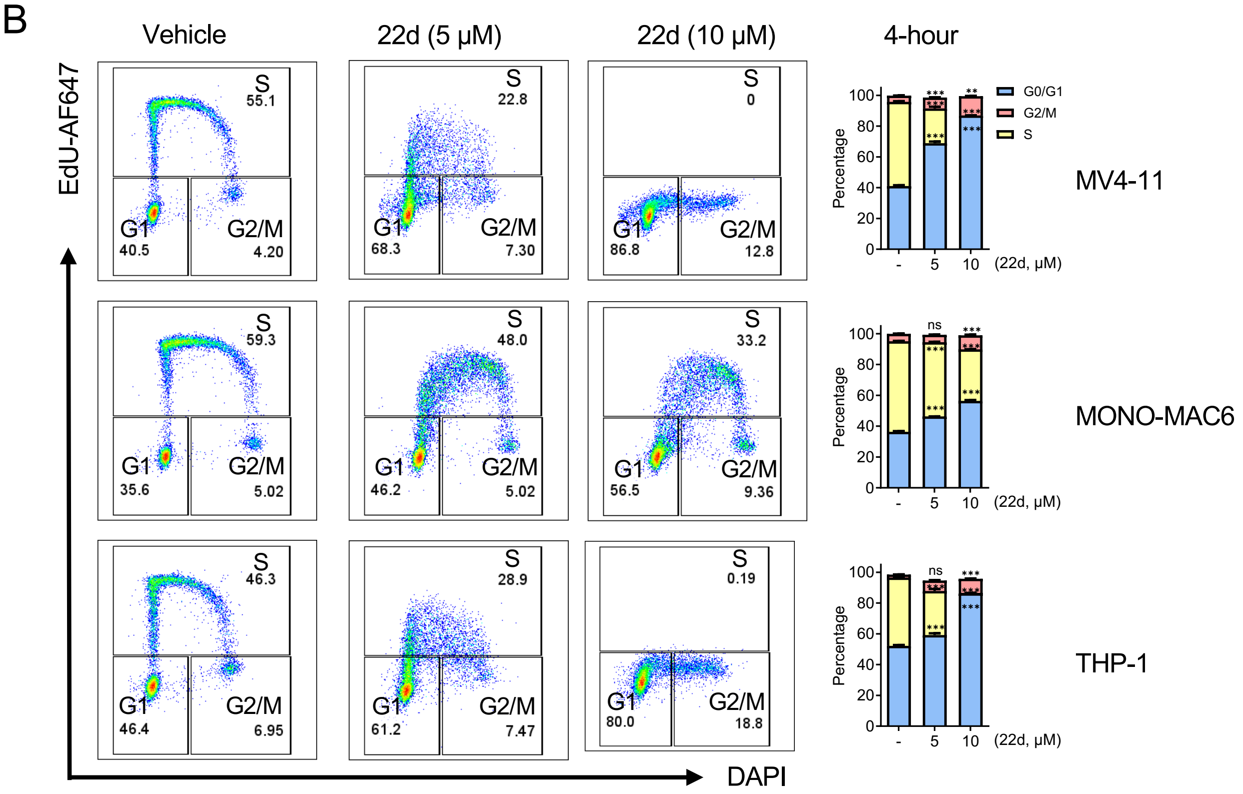

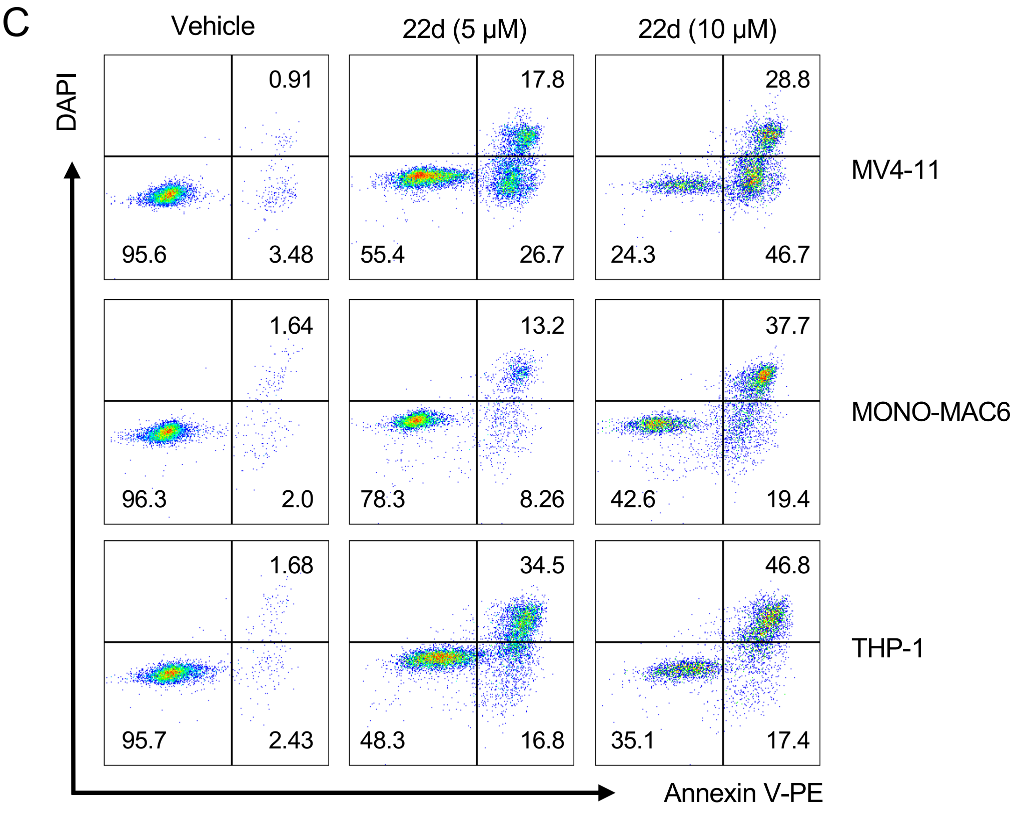

Figure S2. HDAC8i (22d) treatment incudes cell cycle arrest followed by apoptosis in AML cells regardless of *TP53* status.

A. Summary of *TP53* status across AML cell lines used in this study.

B. (Left) Representative flow cytometry plots showing EdU incorporation and cell cycle distribution in MV4-11, MONO-MAC6, and THP-1 cells 4 hours after HDAC8i (22d, 5 or 10 μM) treatment. The percentage of cells in G_1, S, and G_2/M phases are indicated for each condition. (Right) Cell cycle analysis assessed by EdU-PI dual staining in MV4-11, MONO-MAC6 and THP-1 cells treated with vehicle or HDAC8i (22d, 5 or 10 μM) for 4 or 12 hours (n=3). Data are presented as mean ± SEM; Statistical significance was assessed using unpaired test (*p<0.05, **p<0.01, ***p<0.001).

C. Representative plots of apoptosis in MV4-11, MONO-MAC6, and THP-1 cells 24 hours after treatment with HDAC8i (22d, 5 or 10 μM). Cells were stained with Annexin V-PE and DAPI.

Figure S3

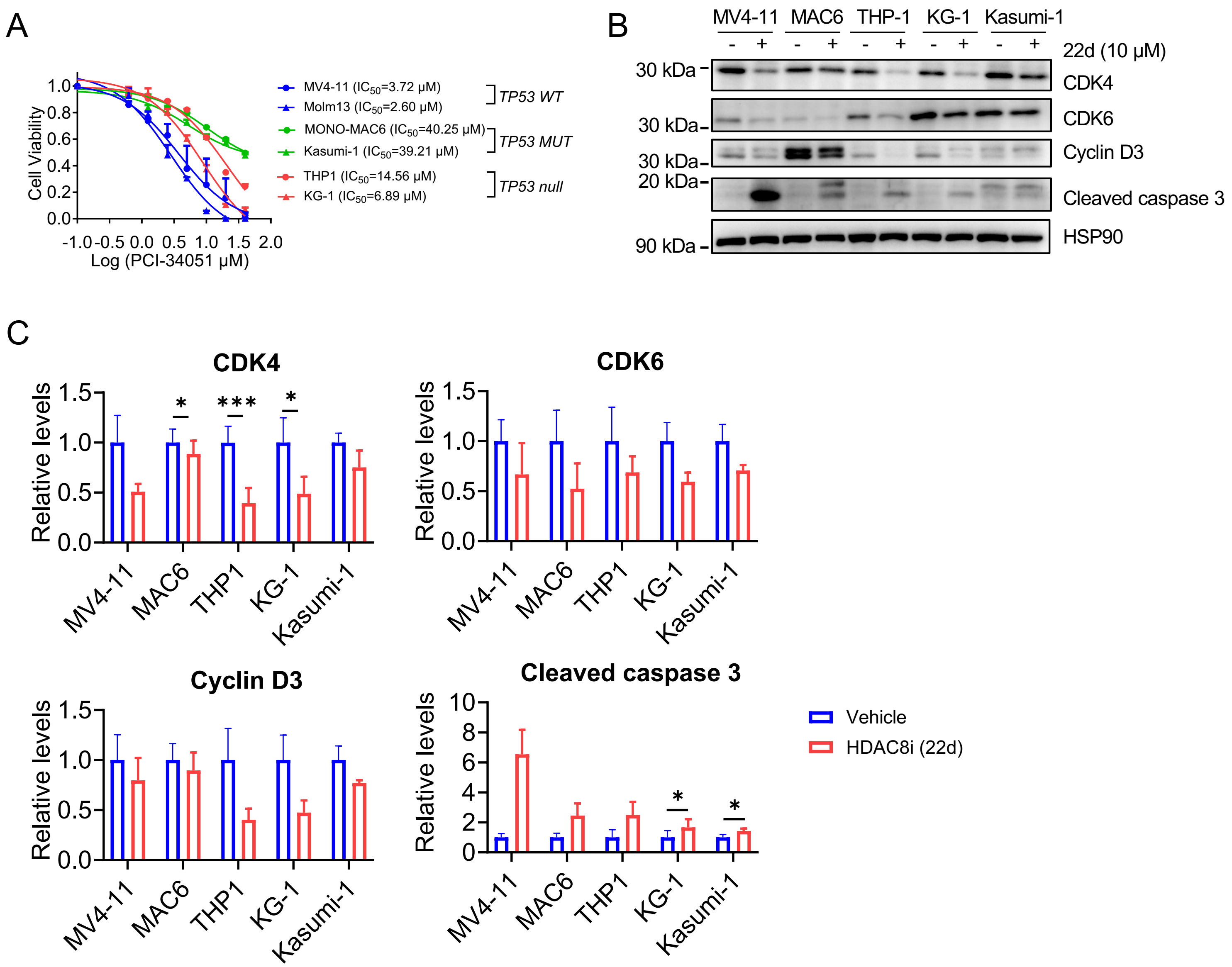

Figure S3. HDAC8 inhibition reduces cell viability and expression of cell cycle regulators in AML cells.

A. Cell viability inhibitory curve and IC_{50} of HDAC8i (PCI-34051) in AML cell lines with *TP53-WT* (MV4-11, Molm-13), *TP53*-MUT (MONO-MAC6, Kasumi-1) or *TP53*-null (THP-1, KG-1) (n=3).

B. Representative immunoblotting (IB) of cell cycle regulators (CDK4, CDK6, Cyclin D3) and cleaved caspase 3 in AML cell lines after treated with HDAC8i (22d) for 24 hours. Representative blot is shown; additional replicates are provided in the Supportive Information.

C. Relative levels of cell cycle regulators (CDK4, CDK6, Cyclin D3) and cleaved caspase 3 in AML cell lines quantified from three independent experiments (n=3).

Data are presented as mean ± SEM; Statistical significance was assessed using t-test (*$p < 0.05$, ***$p < 0.001$).

Figure S4

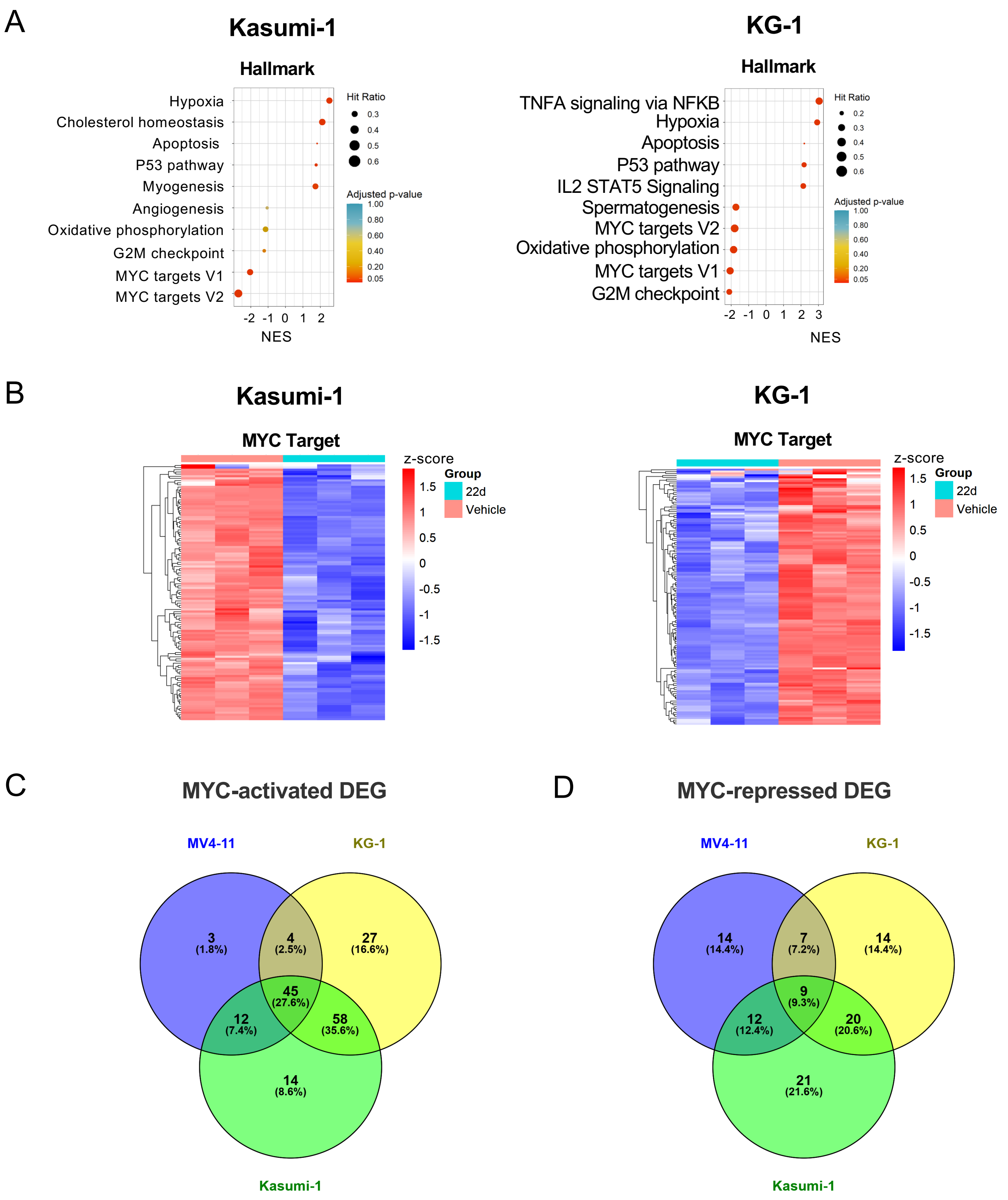
A
Kasumi-1
Hallmark
Hypoxia
Cholesterol homeostasis
Apoptosis
P53 pathway
Myogenesis
Angiogenesis
Oxidative phosphorylation
G2M checkpoint
MYC targets V1
MYC targets V2
-2 -1 0 1 2
NES
Hit Ratio
0.3
0.4
0.5
0.6
Adjusted p-value
1.00
0.80
0.60
0.40
0.20
0.05
KG-1
Hallmark
TNFA signaling via NFKB
Hypoxia
Apoptosis
P53 pathway
IL2 STAT5 Signaling
Spermatogenesis
MYC targets V2
Oxidative phosphorylation
MYC targets V1
G2M checkpoint
-2 -1 0 1 2 3
NES
Hit Ratio
0.2
0.3
0.4
0.5
0.6
Adjusted p-value
1.00
0.80
0.60
0.40
0.20
0.05
B
Kasumi-1
MYC Target
z-score
1.5
1
0.5
0
-0.5
-1
-1.5
Group
22d
Vehicle
KG-1
MYC Target
z-score
1.5
1
0.5
0
-0.5
-1
-1.5
Group
22d
Vehicle
C
MYC-activated DEG
MV4-11
KG-1
3 (1.8%)
4 (2.5%)
27 (16.6%)
45 (27.6%)
12 (7.4%)
58 (35.6%)
14 (8.6%)
Kasumi-1
D
MYC-repressed DEG
MV4-11
KG-1
14 (14.4%)
7 (7.2%)
14 (14.4%)
9 (9.3%)
12 (12.4%)
20 (20.6%)
21 (21.6%)
Kasumi-1

Figure S4. HDAC8i (22d) induced dysregulation of MYC-driven transcription network.

A. GSEA of RNA-seq data from Kasumi-1 and KG-1 cells treated with HDAC8i (22d, 10 μM for 12 hours) vs. vehicle control. Dot plots showed Hallmark pathway enrichment analysis (ranked by NES: highest 5 and lowest 5). Dot size represents the hit ratio, defined as the number of enriched genes in a given pathway divided by the total number of genes in that pathway. The x-axis indicates normalized enrichment scores (NES), and colors denote p-values. Full list is provided in Supplementary Table 9.

B. Heatmap showing gene-wise z-score–normalized expression of leading-edge Hallmark MYC targets in vehicle and HDAC8i (22d) treated Kasumi-1 (119 genes) and KG-1 cells (134 genes), with red indicating high expression and blue indicating low expression.

C. Venn-diagram of MYC-activated genes (Hallmark MYC targets; M5926 and M5928) that are down-regulated (Table S10) in HDAC8i (22d) treated MV4-11, Kasumi-1 and KG-1 cells.

D. Venn-diagram of MYC-repressed genes (DANG MYC TARGETS DN gene set; M2310) that are up-regulated (Table S10) in HDAC8i (22d) treated MV4-11, Kasumi-1 and KG-1 cells.

Figure S5

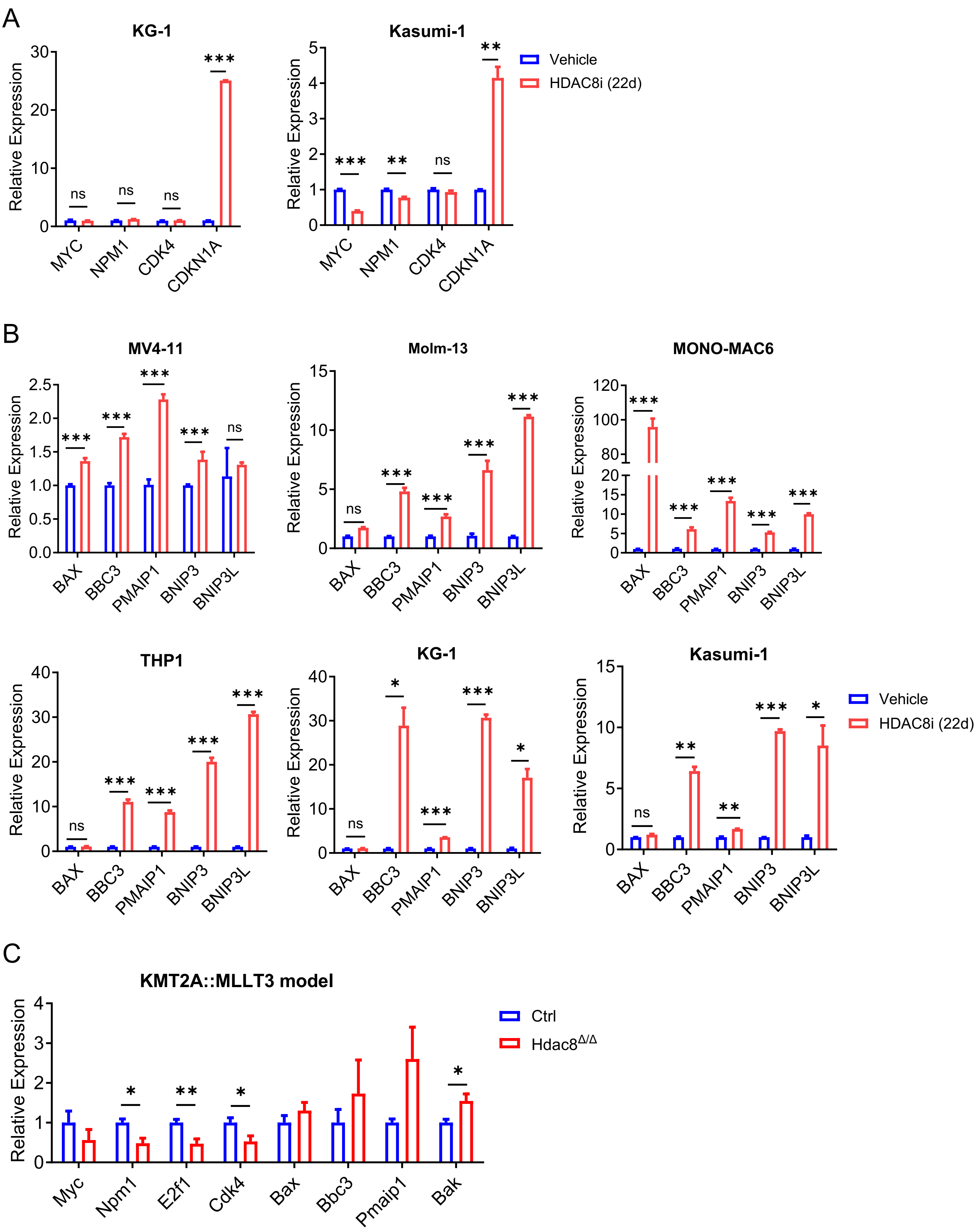
A
KG-1
Kasumi-1
Relative Expression
Vehicle
HDAC8i (22d)
MYC
NPM1
CDK4
CDKN1A
ns
***
**
B
MV4-11
Molm-13
MONO-MAC6
THP1
KG-1
Kasumi-1
BAX
BBC3
PMAIP1
BNIP3
BNIP3L
C
KMT2A::MLLT3 model
Ctrl
Hdac8Δ/Δ
Myc
Npm1
E2f1
Cdk4
Bax
Bbc3
Pmaip1
Bak

Figure S5. Dysregulation of MYC targets and pro-apoptotic genes by pharmacological inhibition or genetic deletion of HDAC8 in AML cells.

A. Relative expression of *MYC*, *NPM1*, *CDK4*, and *CDKNA1* measured by qPCR analysis in KG-1, and Kasumi-1 AML cells treated with vehicle or HDAC8i (22d, 10 μM, 24 hours). *ACTB* was used as an internal control.

B. Relative expression of *BAX*, *BBC3*, *PMAIP1*, *BNIP3*, and *BNIP3L* measured by *q*PCR analysis in MV4-11, Molm-13, MONO-MAC6, THP-1, KG-1, and Kasumi-1 AML cells treated with vehicle or HDAC8i (22d, 10 μM, 24 hours). *ACTB* was used as an internal control.

C. Relative expression of *Myc* targets and pro-apoptosis genes by qPCR analysis in murine *KMT2A::MLLT3 AML* model with genetic *Hdac8* deletion. *B2m* was used as internal control.

Data are presented as mean ± SEM; Statistical significance was assessed using unpaired test (***p < 0.001).

Figure S6

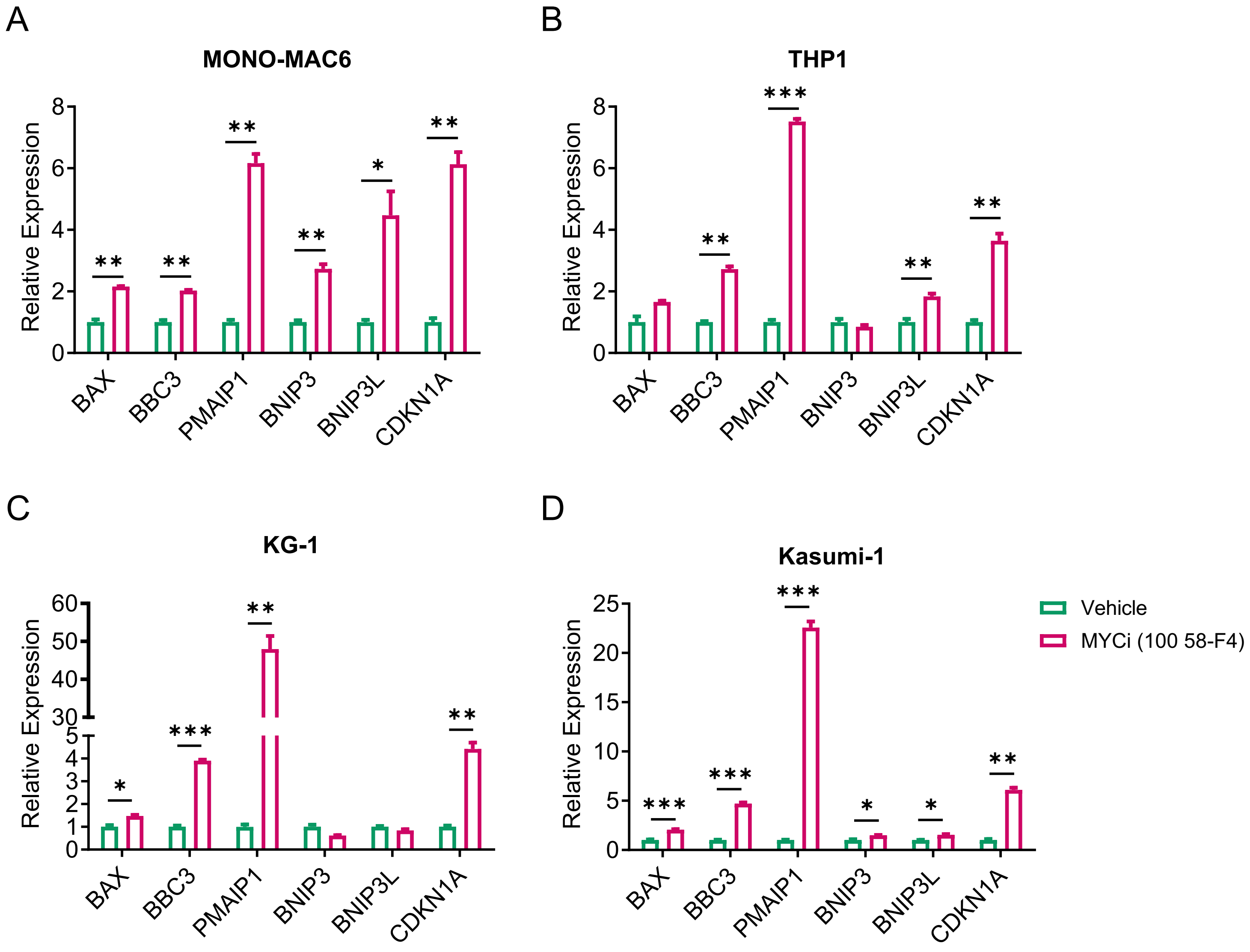

Figure S6. Analysis of cell cycle and pro-apoptotic genes in AML cells treated with MYC inhibitor (10058-F4).

A-D. Relative expression of *BAX*, *BBC3*, *PMAIP1*, *BNIP3*, *BNIP3L,* and *CDKN1A* measured by qPCR analysis in MONO-MAC6, THP-1, KG-1 and Kasumi-1 AML cells treated with vehicle or MYC inhibitor (10058-F4, 100 µM, 24 hours). *ACTB* was used as an internal control. Data are presented as mean ± SEM; Statistical significance was assessed using unpaired test (*p < 0.05, **p < 0.01 ***p < 0.001).

Figure S7

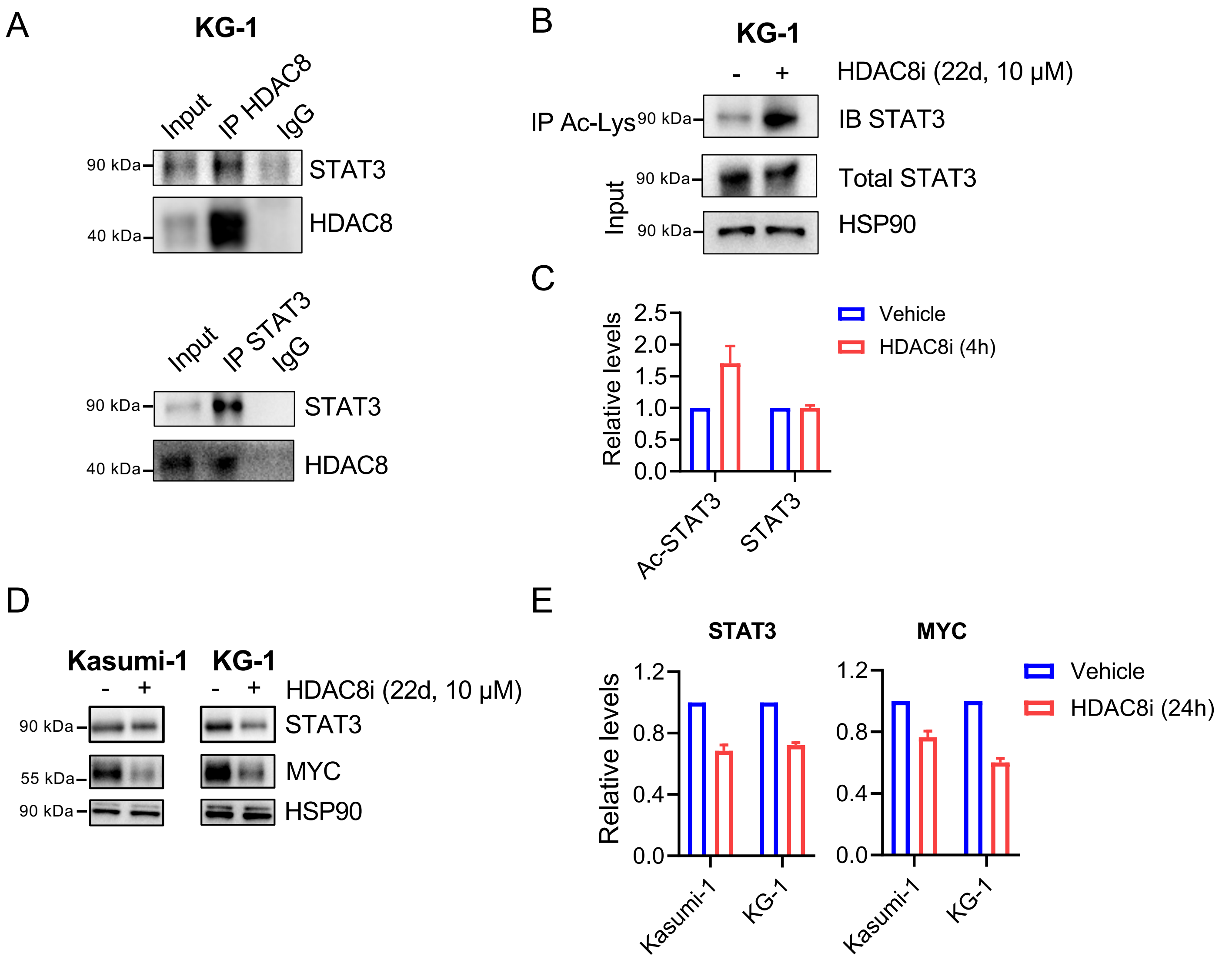

Figure S7. HDAC8 physically interacts with STAT3, deacetylates STAT3 and regulate MYC expression.

A. Representative co-IP using anti-HDAC8 (top) or anti-STAT3 (bottom) antibodies followed by IB to detect HDAC8 and STAT3 interaction in KG-1 cells. Representative blot is shown; additional replicates are provided in the Supportive Information.

B. Representative co-IP with anti-acetylated-Lysine (Ac-Lys) antibody followed by IB to detect acetylated (Ac)-STAT3 and IB for total STAT3, MYC in KG-1 cells treated with HDAC8i (22d) or vehicle. Representative blot is shown; additional replicates are provided in the Supportive Information.

C. Relative levels of Ac-STAT3 and total STAT3 protein quantified from three independent experiments.

D-E. Representative immunoblotting (D) and quantification (E) of STAT3 and MYC protein levels in Kasumi-1 and KG-1 cell line treated with vehicle or HDAC8i (22d, 10 μM, 24 hours). Representative blot is shown; additional replicates are provided in the Supportive Information.

Figure S8

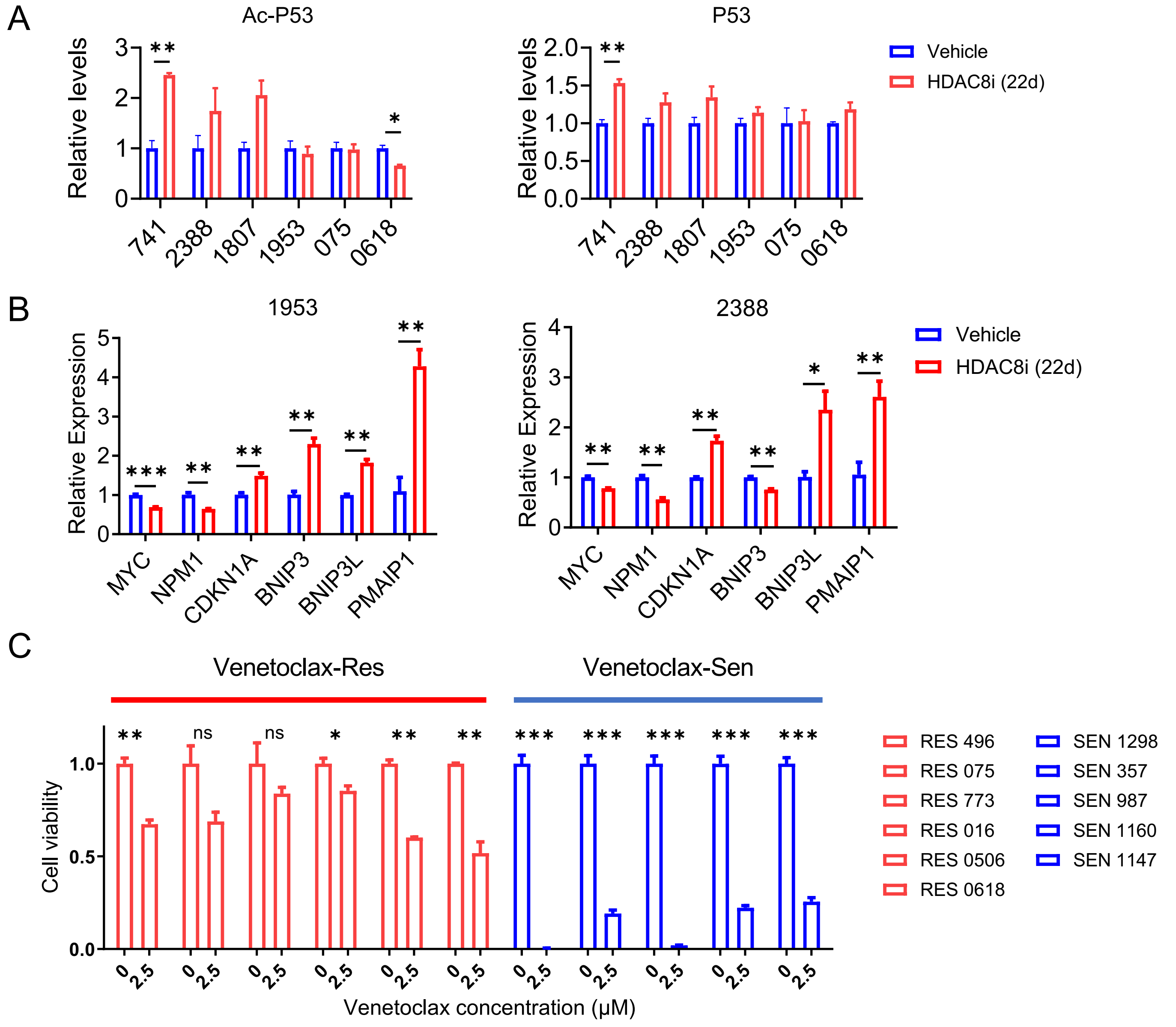

Figure S8. Quantification of protein/gene expression and cell viability assays in AML patient samples.

A. Quantification of protein levels (Ac-P53, P53) in primary AML samples after 24-hour treatment with HDAC8i (22d, 10 μM) or vehicle.

B. Relative mRNA expression levels for *MYC, NPM1, CDK4, P21, BNIP3, BNIP3L,* and *PMAIP1* measured by qPCR analysis in *TP53* mutant (1953) AML patient sample or *TP53* wild-type (2388) AML patient sample 24 hours after treatment with HDAC8i (22d, 10 μM) or vehicle. *ACTB* was used as an internal control.

C. Relative cell viability of AML samples treated with 0 μM or 2.5 μM venetoclax. Blue bars represent venetoclax-sensitive samples (AML1298, 357, 987, 1160, 1147), and red bars represent relatively resistant samples (AML 496, 075, 773, 016, 0506, 0618).

Data are presented as mean ± SEM; statistical significance was assessed using unpaired t-test (*p < 0.05, **p < 0.01, ***p < 0.001).

Figure S9

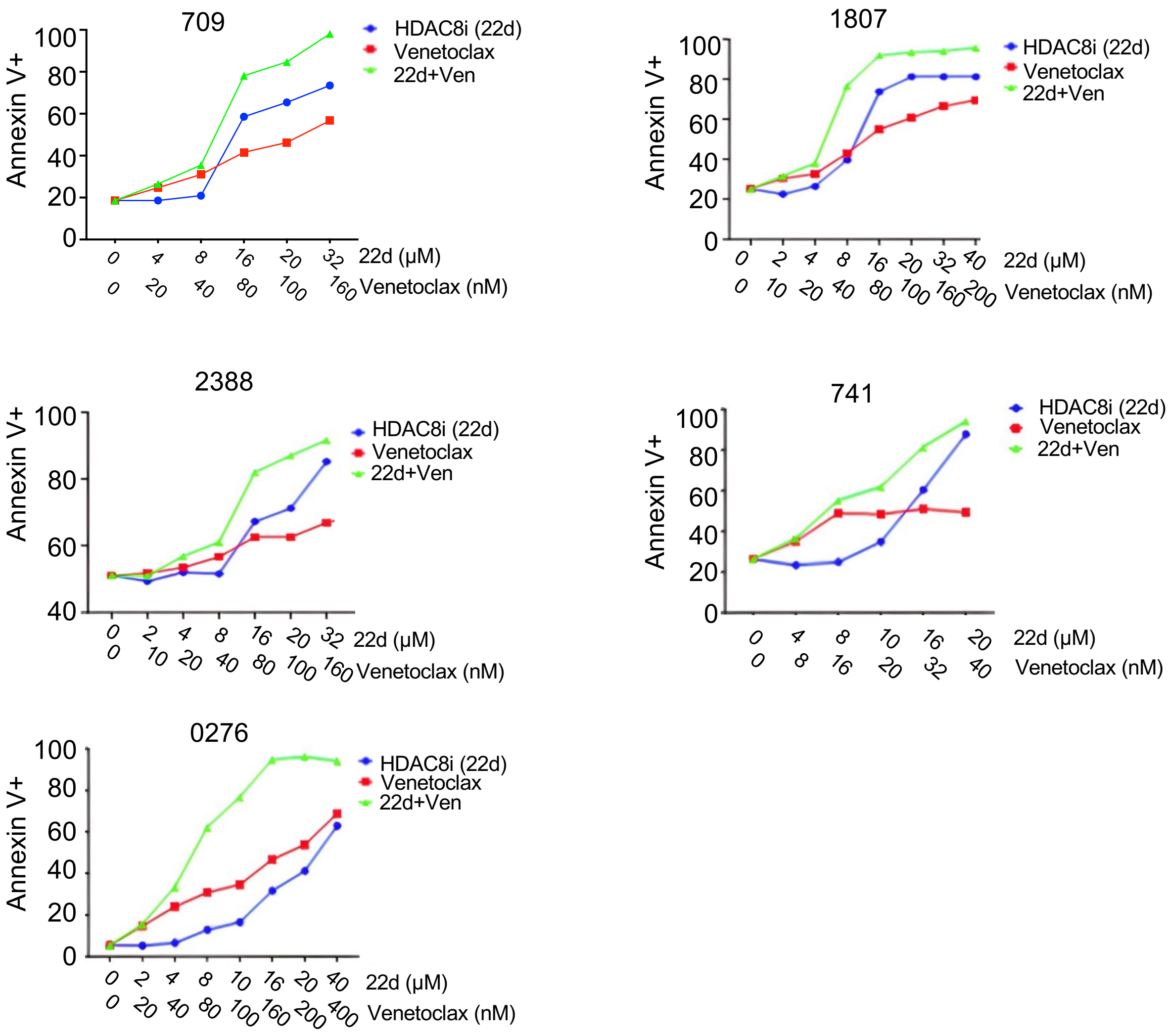

Figure S9. Apoptosis rates in primary AML cells treated with HDAC8i (22d), Venetoclax or the combination.

The frequency of apoptosis (%) in six AML patients' cells treated with HDAC8i (22d; blue line), Venetoclax (red line), and the combination (green line) at specified concentrations.

Figure S10

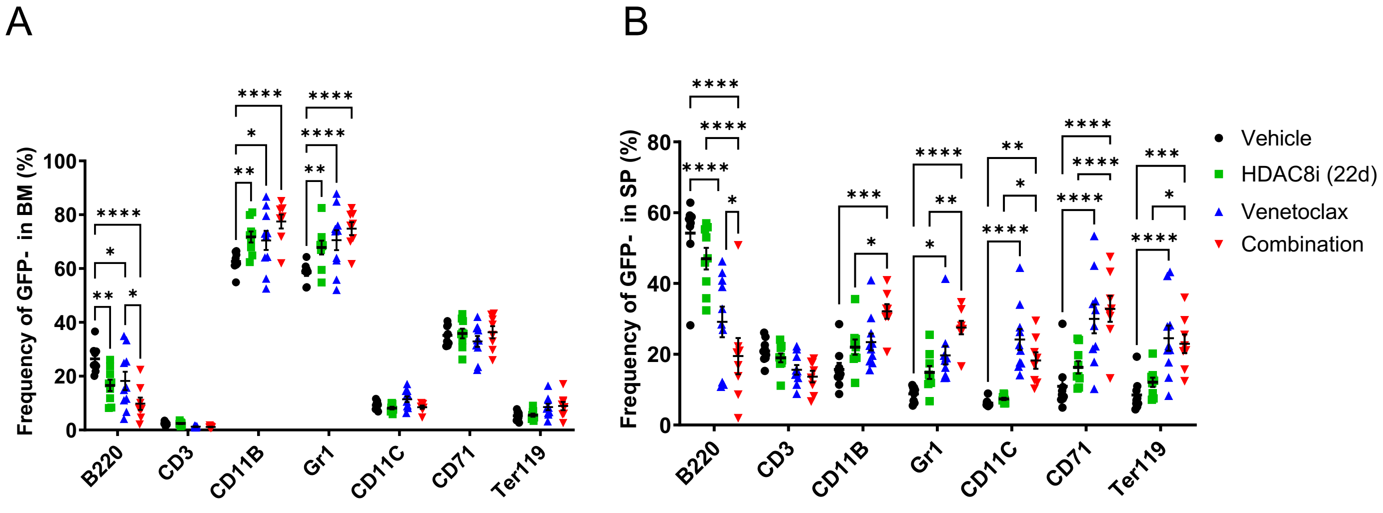

Figure S10. Effects of HDAC8i (22d), venetoclax or combination treatment on the non-leukemic immune cell populations.

The frequency of phenotypic mature cell populations in the non-leukemic (GFP^-) BM compartment (A) or spleen (B, SP) of mice treated with vehicle (n=9), HDAC8i (22d, n=9), Venetoclax (n=10) or the combination (n=8).

Data are shown as mean + SEM. Significance of each comparison was determined using two-way ANOVA tests (*p< 0.05, **p < 0.01, ***p < 0.001, ****p < 0.0001).

Figure S11

A

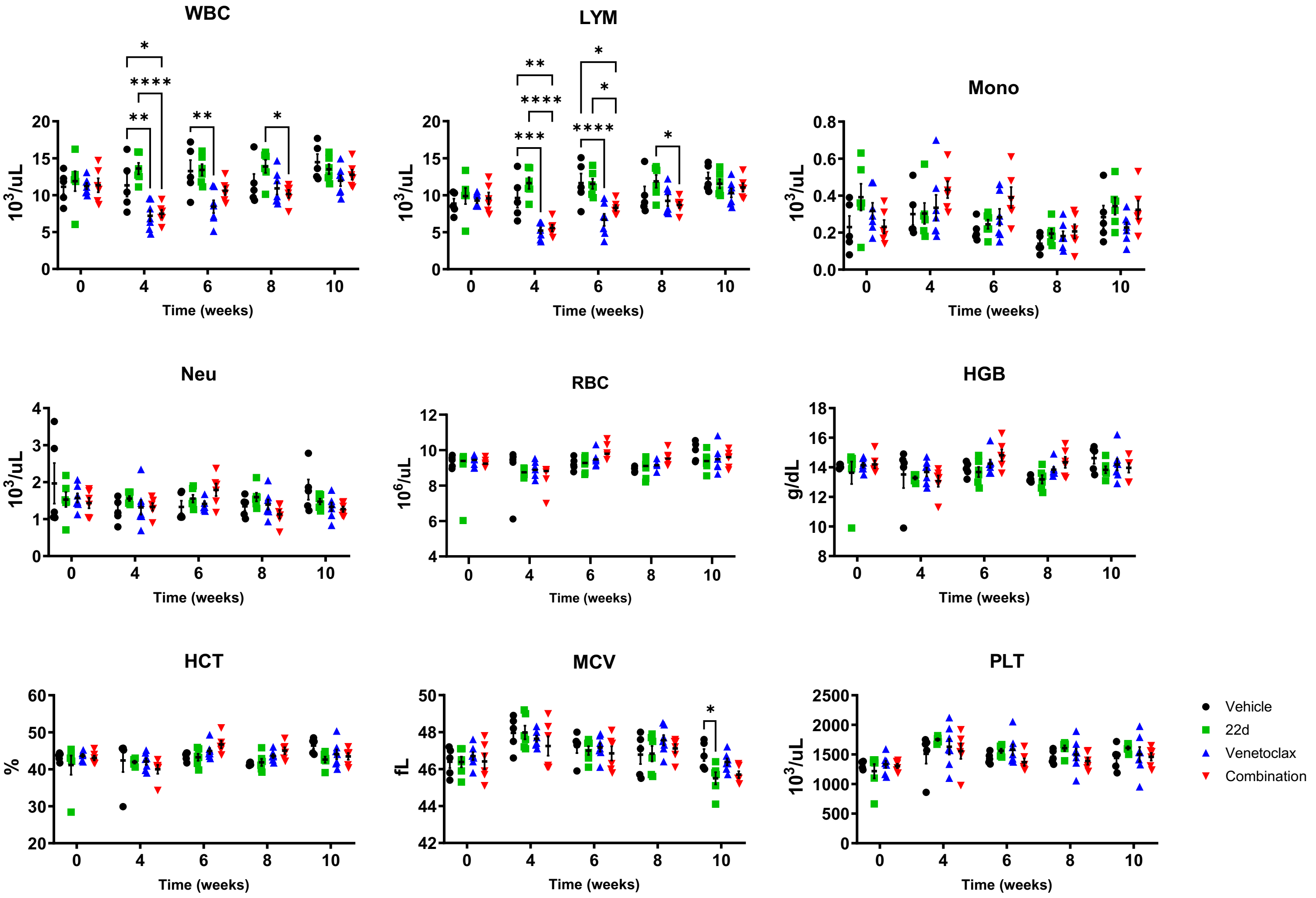

B

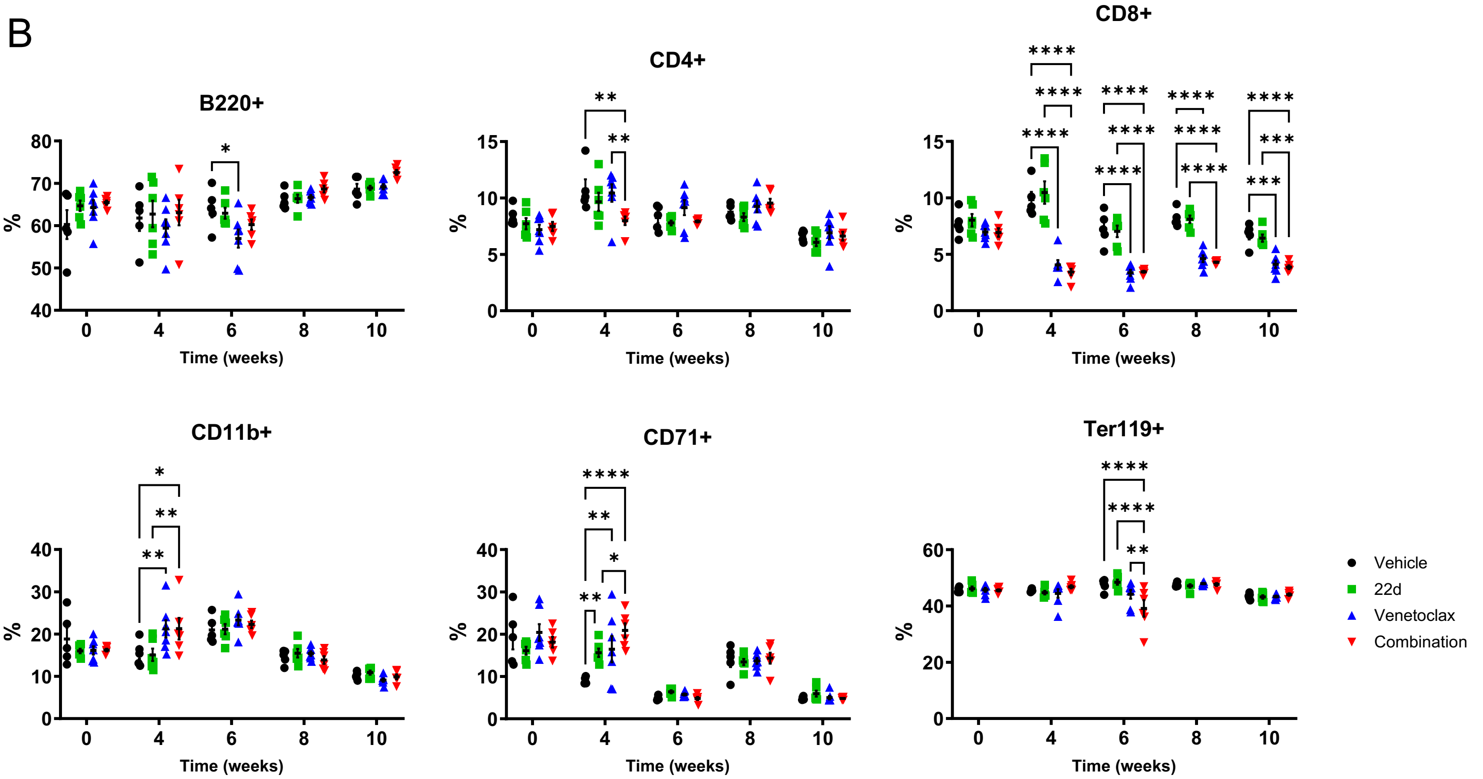

Figure S11. Evaluation of effects of HDAC8i (22d), venetoclax or combination treatment on normal hematopoiesis over time in peripheral blood.

A. Complete blood count analysis of peripheral blood including white blood cells (WBC), lymphocytes (LYM), monocytes (MONO), neutrophils (NEU), red blood cells (RBC), hemoglobin (HGB), hematocrit (HCT), mean corpuscular volume (MCV), platelets (PLT) from WT C57BL6 mice over time (0, 4, 6, 8, 10 weeks) after treatment with vehicle, HDAC8i (22d), Venetoclax, or the combination for 2 weeks.
B. The frequency of phenotypic immune cell populations in the peripheral blood of WT C57BL6 mice over time (0, 4, 6, 8, 10 weeks) after treatment with vehicle, HDAC8i (22d), Venetoclax, or the combination for 2 weeks.

Each dot represents data from an individual mouse. Data are shown as mean + SEM. Significance of each comparison was determined using two-way ANOVA tests (*$p< 0.05$, **$p < 0.01$, ***$p < 0.001$, ****$p < 0.0001$).

Figure S12

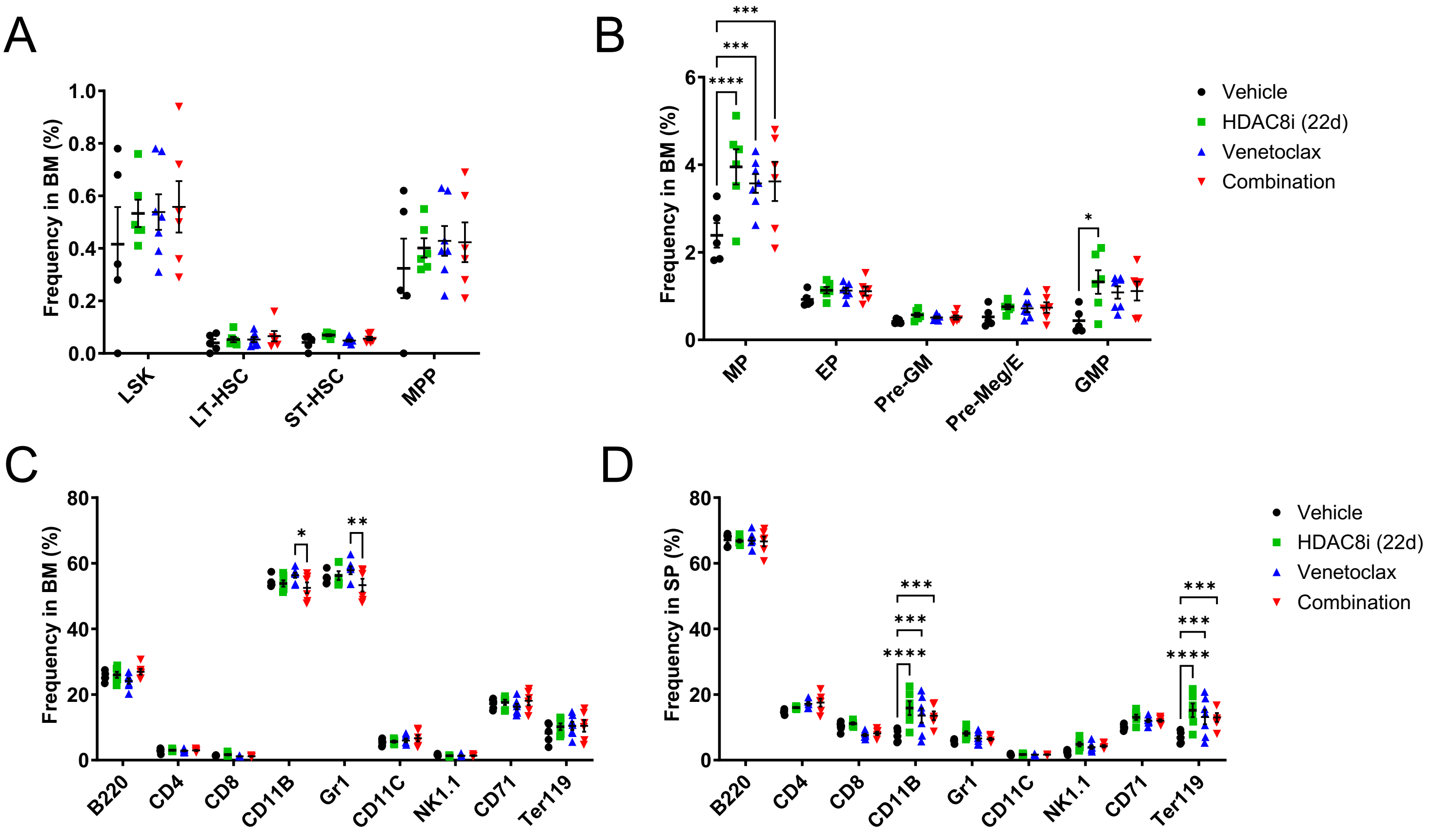

Figure S12. Effects of HDAC8i (22d), venetoclax, and combination therapy on normal hematopoietic immunophenotypic subsets.

A-B. The frequency of phenotypic hematopoietic stem and progenitor populations, including LSK (Lin−cKit+Sca1+), long-term HSCs (LT-HSC; Lin−cKit+Sca1+CD48−CD150+), short-term HSCs (ST-HSC; Lin−cKit+Sca1+CD48−CD150−), multipotent progenitors (MPP; Lin−cKit+Sca1+CD48+CD150+/−), myeloid progenitors (MPs; Lin−ckit+Sca1−), pre-granulocyte–macrophage (Pre-GM; Lin−ckit+Sca1−CD16/32−/loCD105−CD150−), granulocyte–macrophage progenitors (GMP; Lin−ckit+Sca1−CD16/32+CD150−), pre-megakaryocyte/erythrocyte (Pre-Meg/E; Lin−ckit+Sca1−CD16/32−/loCD105−CD150+), and erythroid progenitors (EP; Lin−ckit+Sca1−CD16/32−/loCD105+) in the bone marrow of WT mice 10 weeks after treatment with vehicle (n = 5), HDAC8 (22d) (n = 6), Venetoclax (n = 7) or the combination (n = 6).

C-D. The frequency of mature hematopoietic lineage populations, including B cells (B220), T cells (CD4, CD8), NK cells (NK1.1), myeloid cells (CD11b, Gr-1), and erythroid cells (CD71, Ter119), in the bone marrow (C) and spleen (D, SP) of WT mice 10 weeks after treatment with vehicle (n = 5), HDAC8 inhibitor (22d) (n = 6), venetoclax (n = 7), or the combination (n = 6).

Each dot represents data from an individual mouse. Data are shown as mean + SEM. Significance of each comparison was determined using two-way ANOVA tests (*p< 0.05, **p < 0.01, ***p < 0.001, ****p < 0.0001).

Figure S13

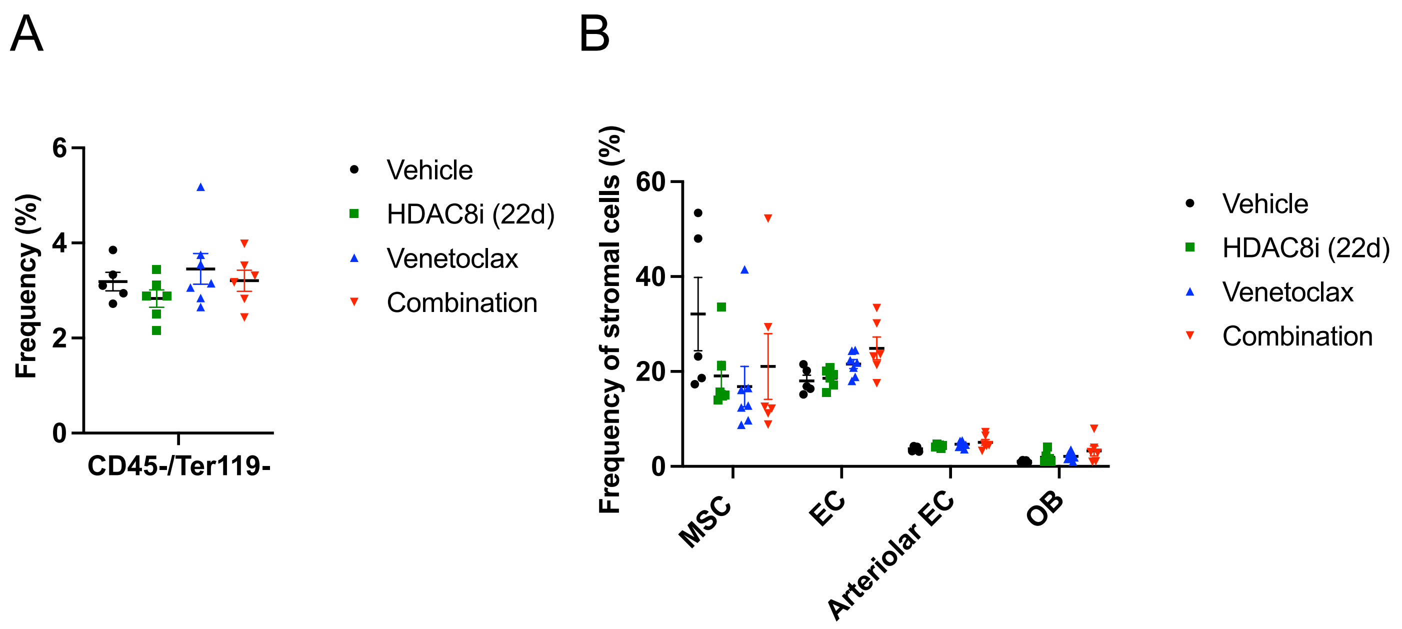

Figure S13. Effects of HDAC8 inhibitor (22d), venetoclax, and combination therapy on normal bone marrow stromal niche populations.

A. The frequency of non-hematopoietic stromal cell populations (CD45-/Ter119-) in the bone marrow of WT mice 10 weeks after treatment with vehicle (n = 5), HDAC8 (22d) (n = 6), Venetoclax (n = 7) or the combination (n = 6).

B. The composition of phenotypic stromal cell populations described in (A). The phenotypic cell populations are defined as follows: mesenchymal stromal cells (MSC; CD45-/Ter119-/CD31-/CD105+/CD271+/Sca1-); endothelial cells (EC; CD45-/Ter119-CD31+); arteriolar EC (CD45-/Ter119-/CD31+/Sca1+); osteoblasts (OB; CD45-/Ter119-/CD166+/Tie2-).

Each dot represents data from an individual mouse. Data are shown as mean + SEM.

Figure S14

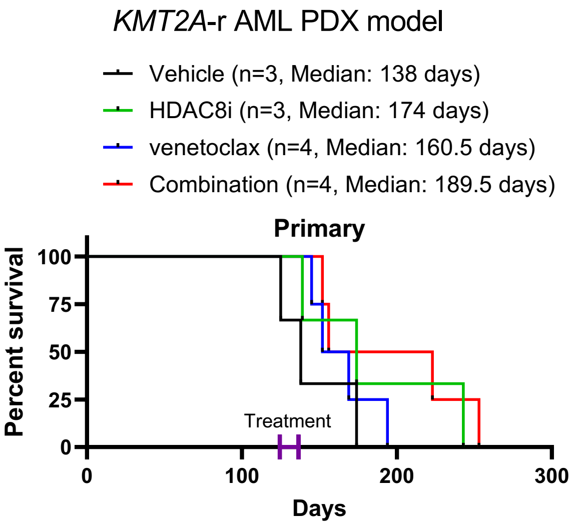

Figure S14. Primary survival curve of human *KMT2A*-r AML PDX model treated with HDAC8i, Venetoclax or the combination.

Kaplan–Meier survival curves of primary KMT2A-r AML 741 PDX mice treated with vehicle (n = 3), HDAC8i (22d; n = 3), Venetoclax (n = 4), or the combination (n = 4).

Statistical significance was assessed using a log-rank (Mantel–Cox) test.
