# Supplementary material for: HDAC8 inhibition targets STAT3–MYC axis and synergizes with Venetoclax in KMT2A-rearranged acute myeloid leukemia
Source: Leukemia. 2026 Apr 21;40(6):1271–83. doi: 10.1038/s41375-026-02950-1 (PMC13233295; doi:10.1038/s41375-026-02950-1)
Supplement: Supplementary file 7 — Supportive Data [file 41375_2026_2950_MOESM7_ESM.pdf]

Supportive Data

Figure 3K

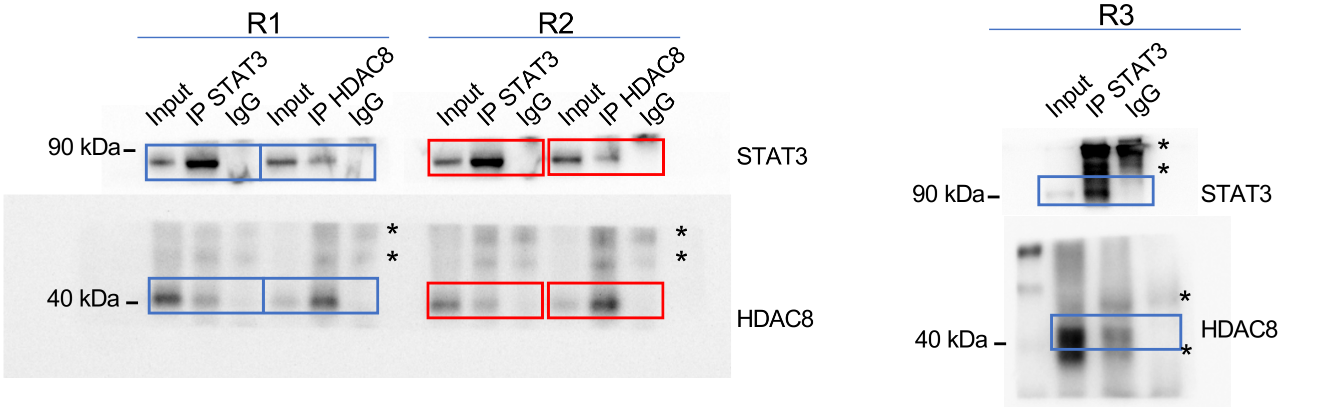

Figure 3L

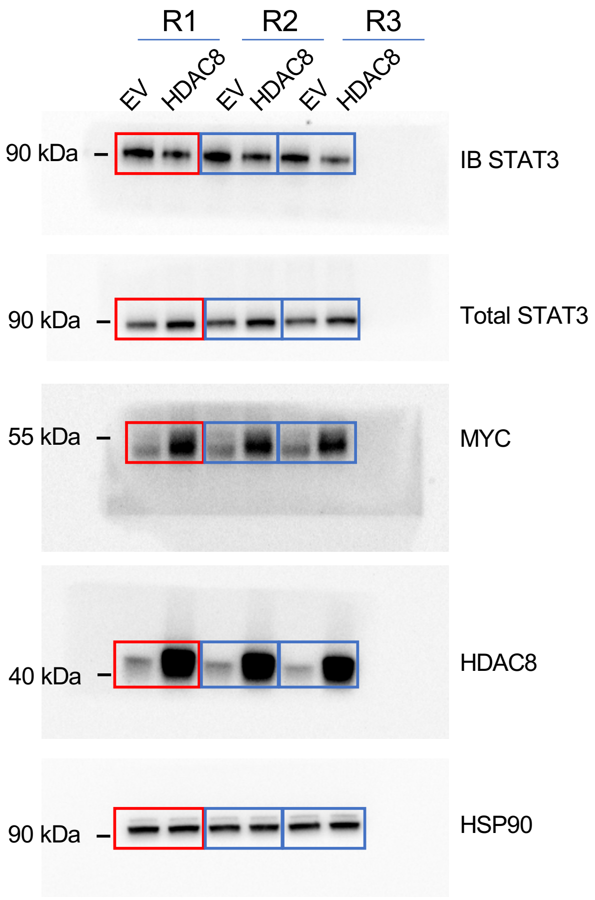

Figure 3N

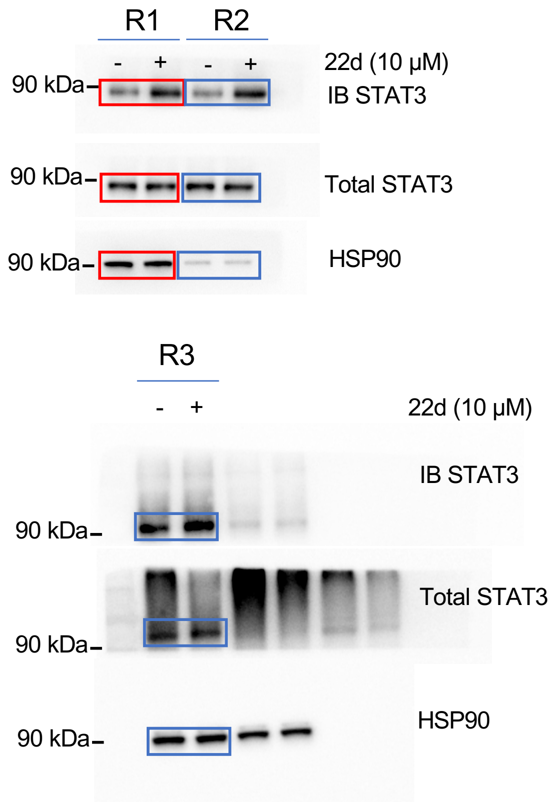

* non-specific band

Supportive Data

Figure 3P

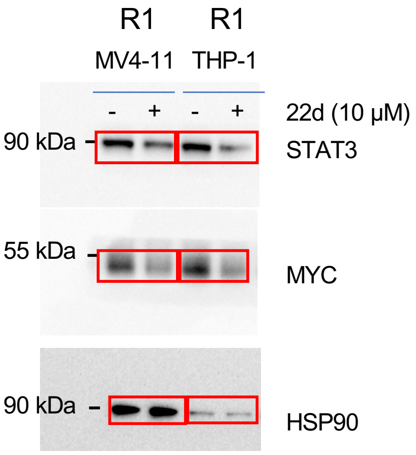

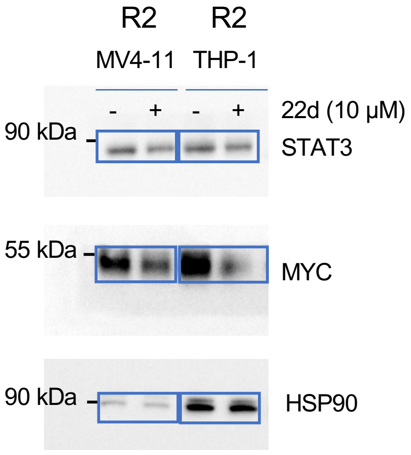

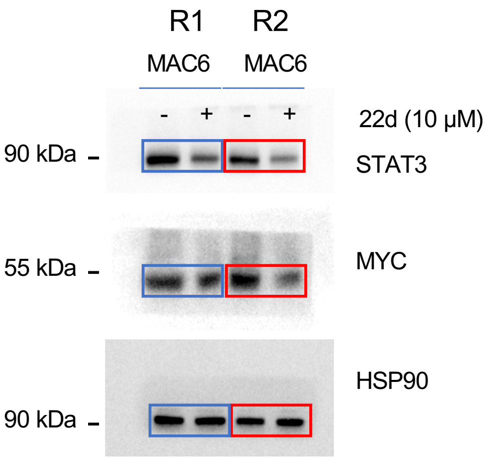

Figure S5D

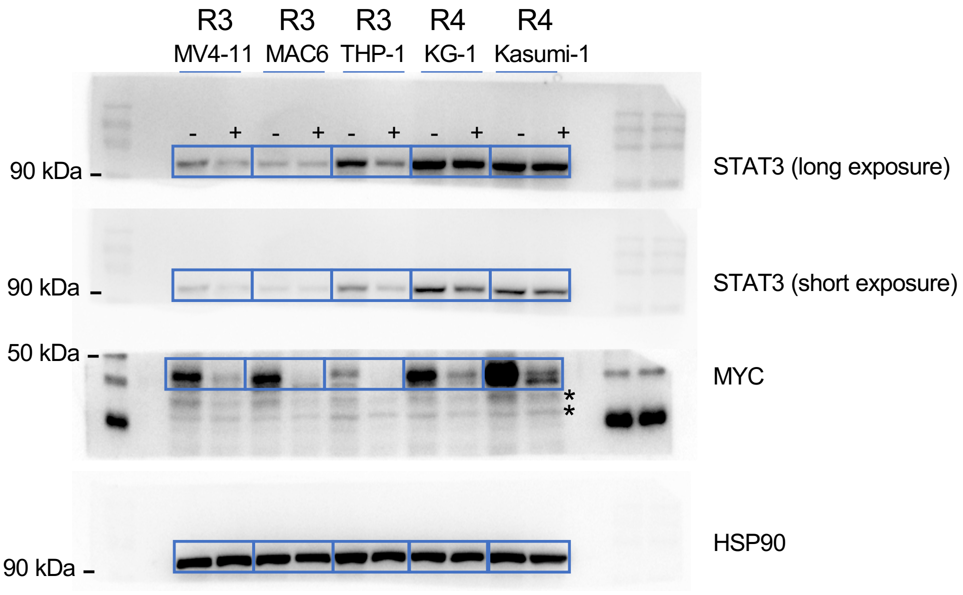

* non-specific band

Supportive Data

Figure 3R

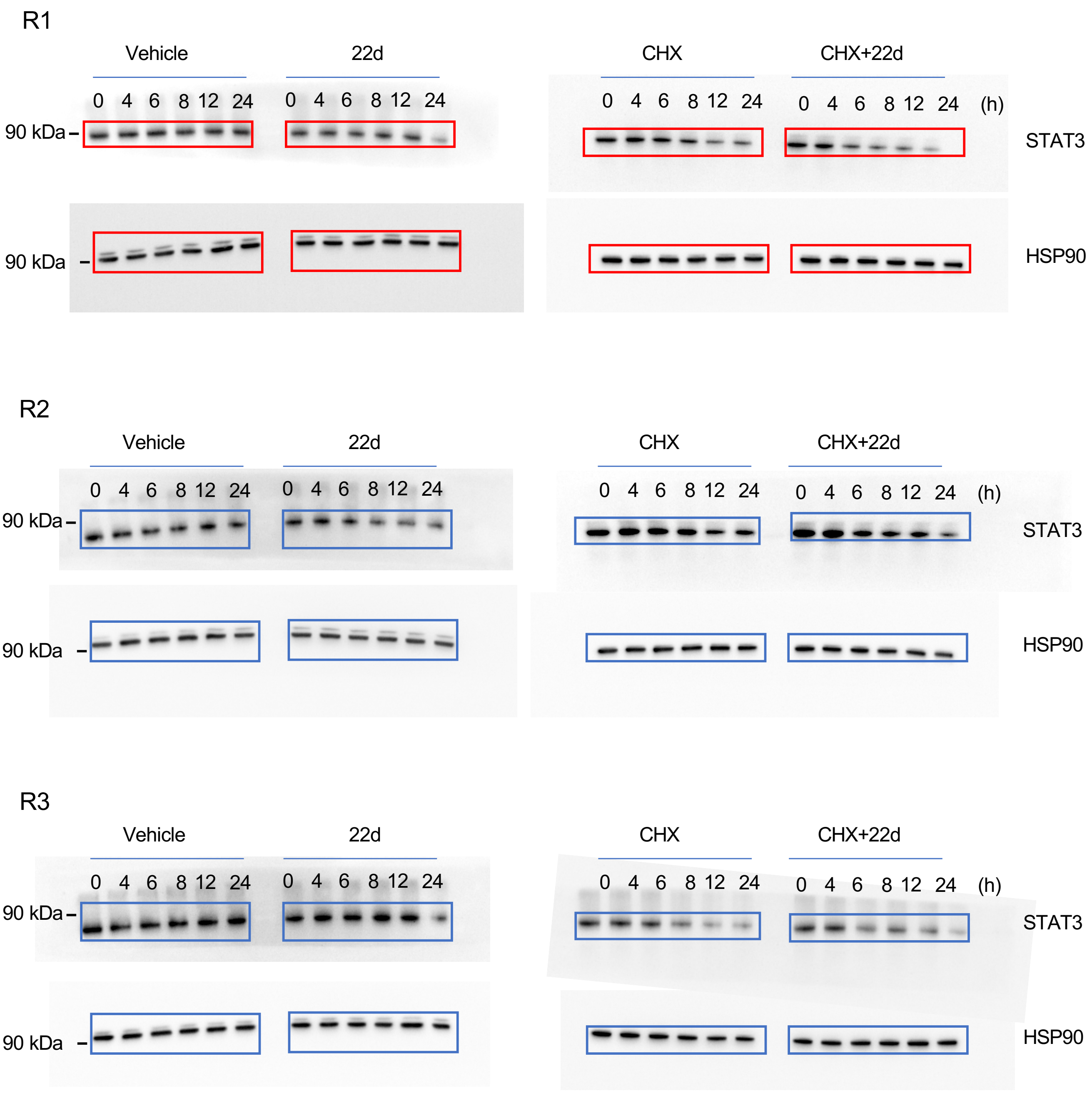

Supportive Data

Figure 4C

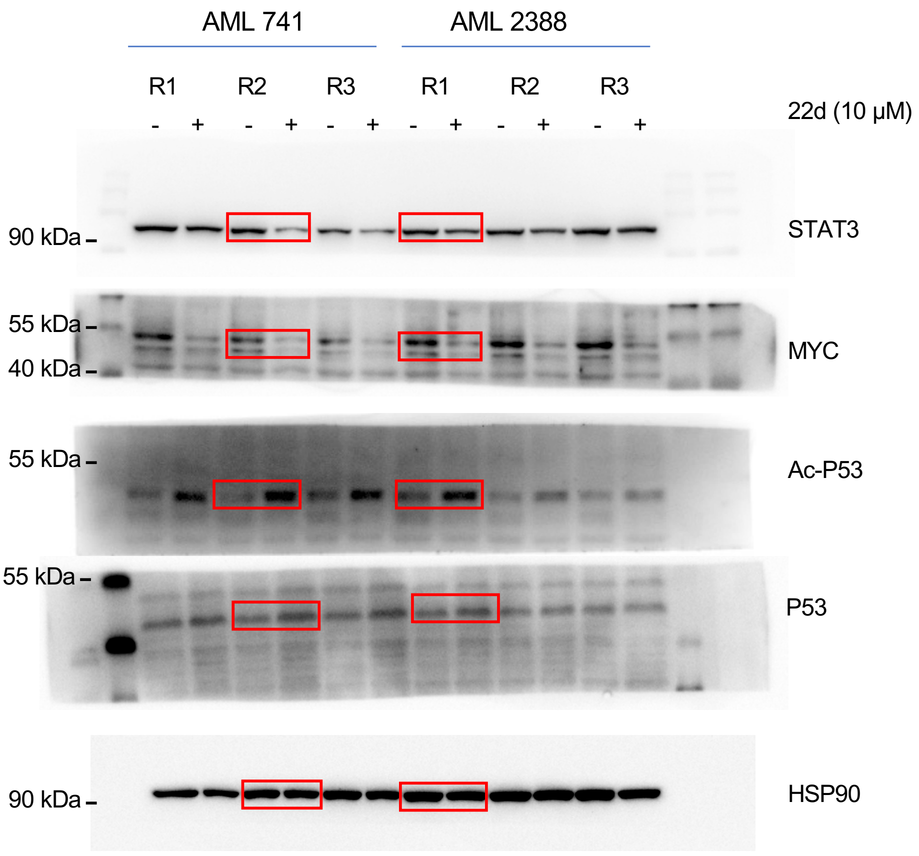

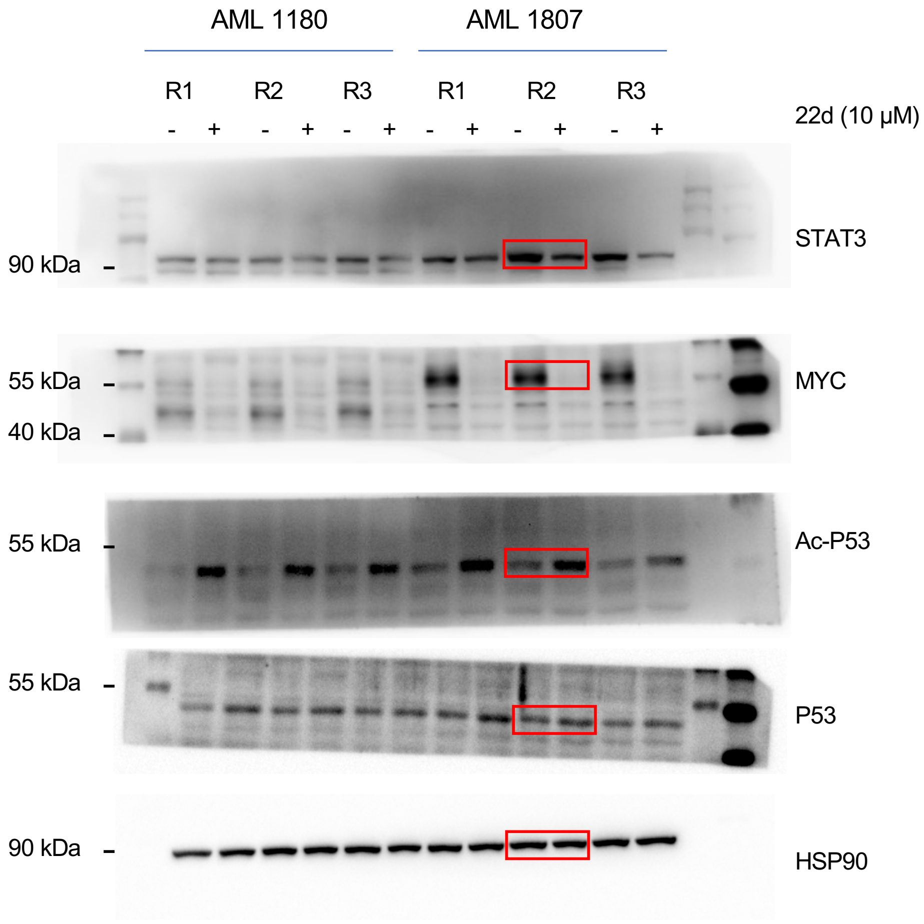

Supportive Data

Figure 4C continued

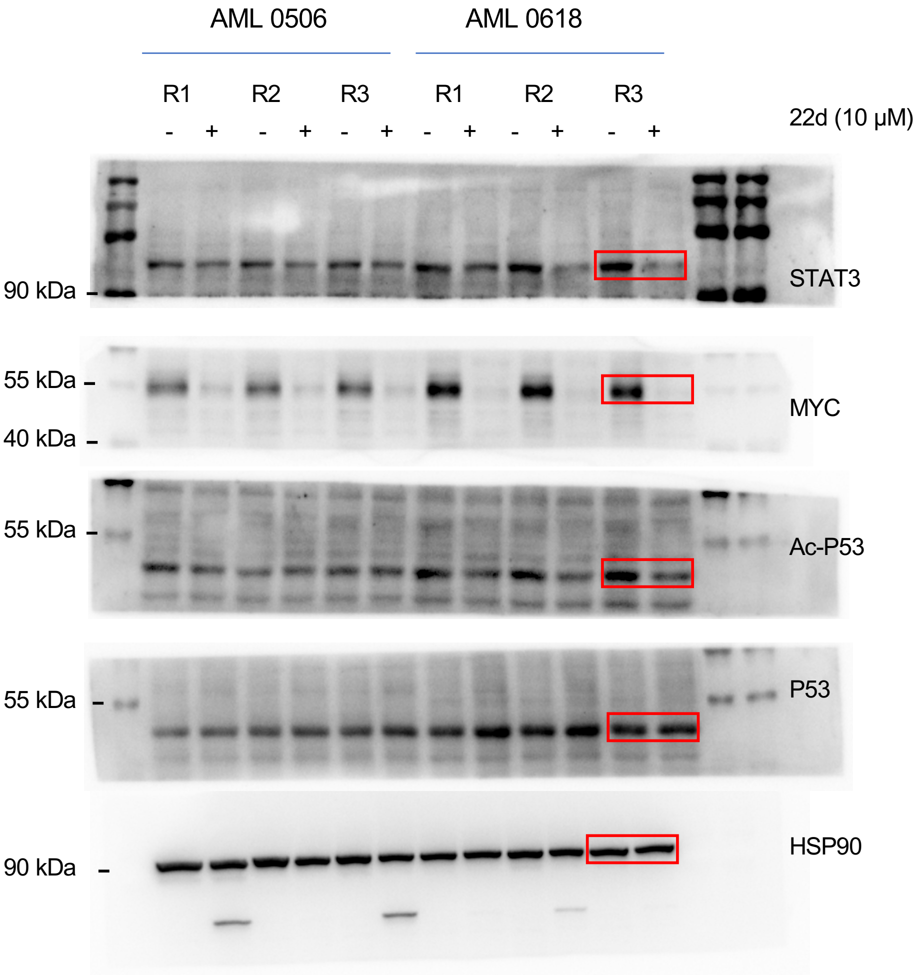

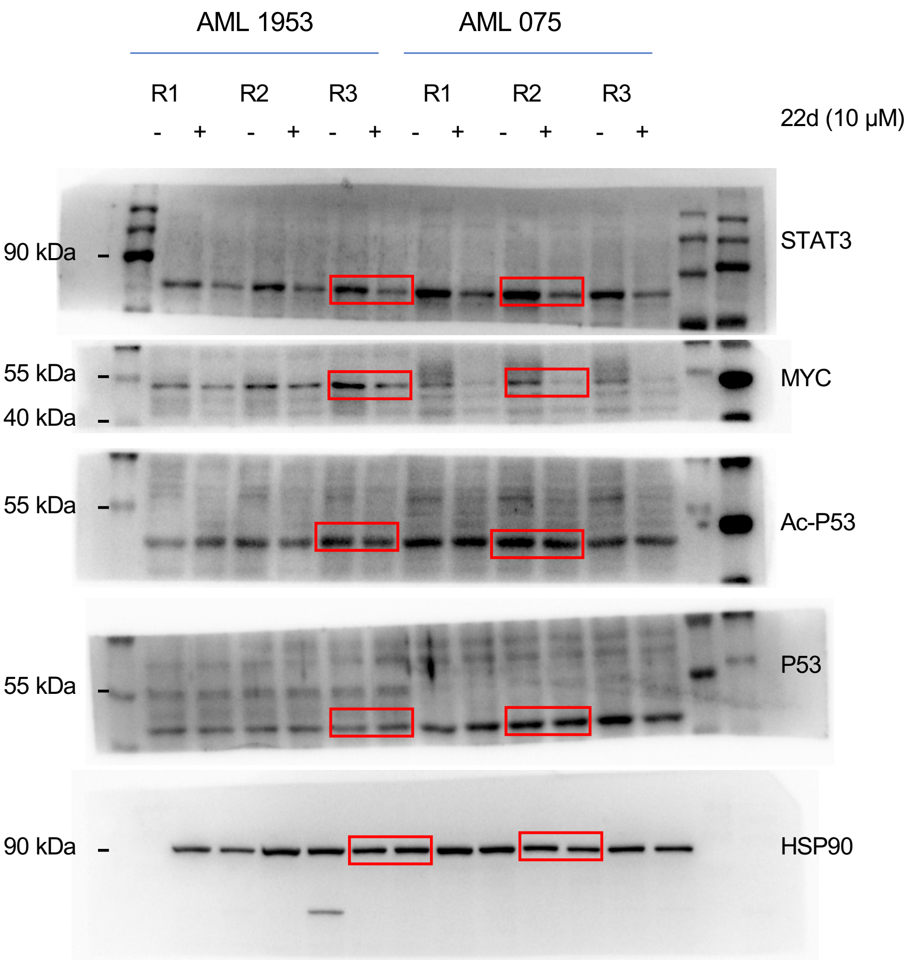

Supportive Data

Figure S3B

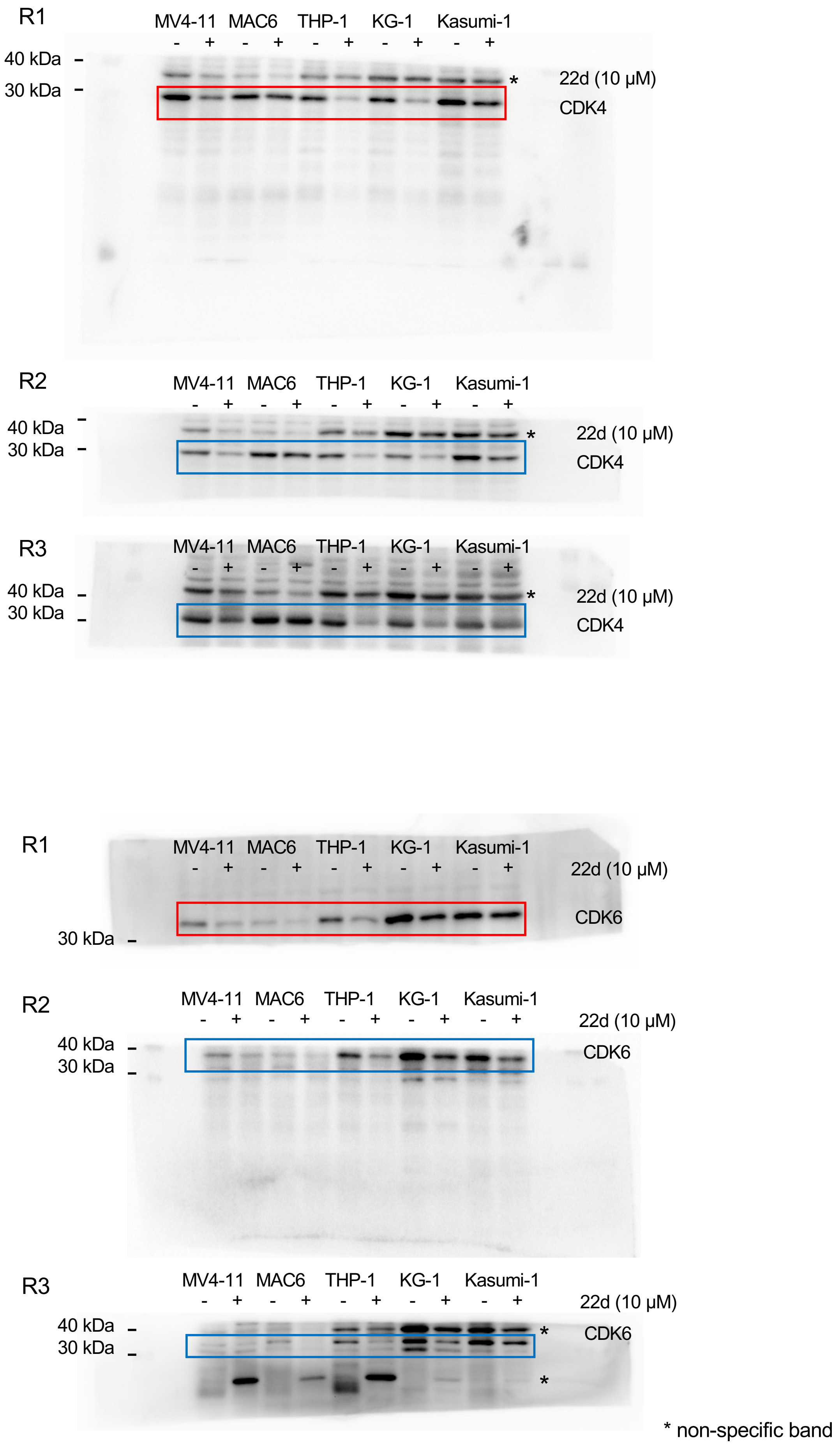

Supportive Data

Figure S3B continued

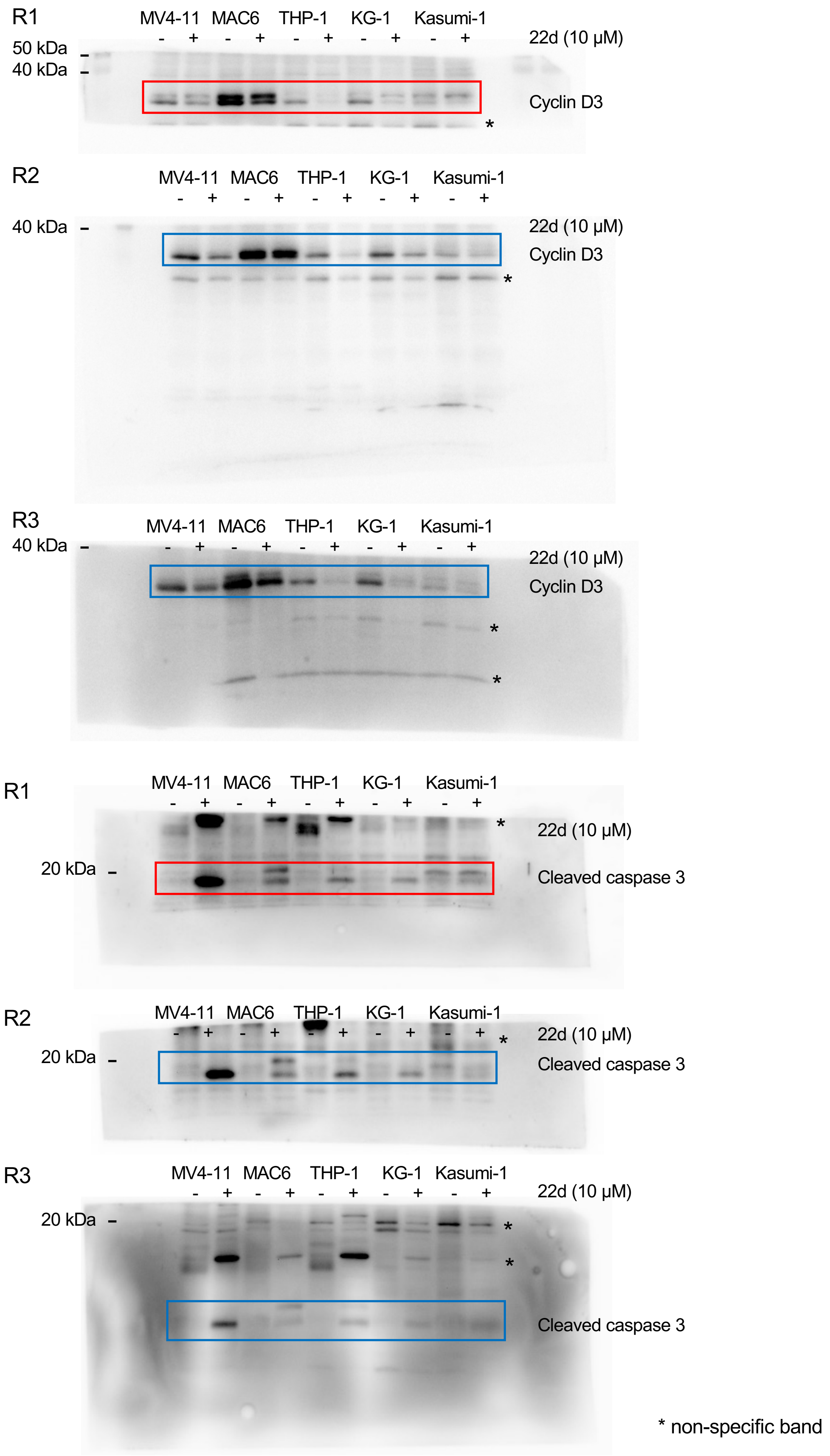

Supportive Data

Figure S3B continued

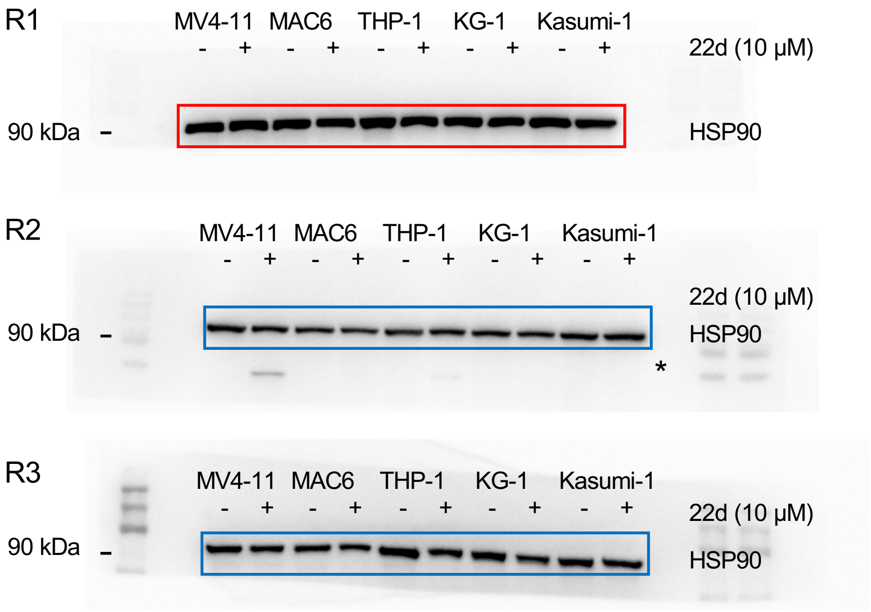

* non-specific band

Supportive Data

Figure S7A

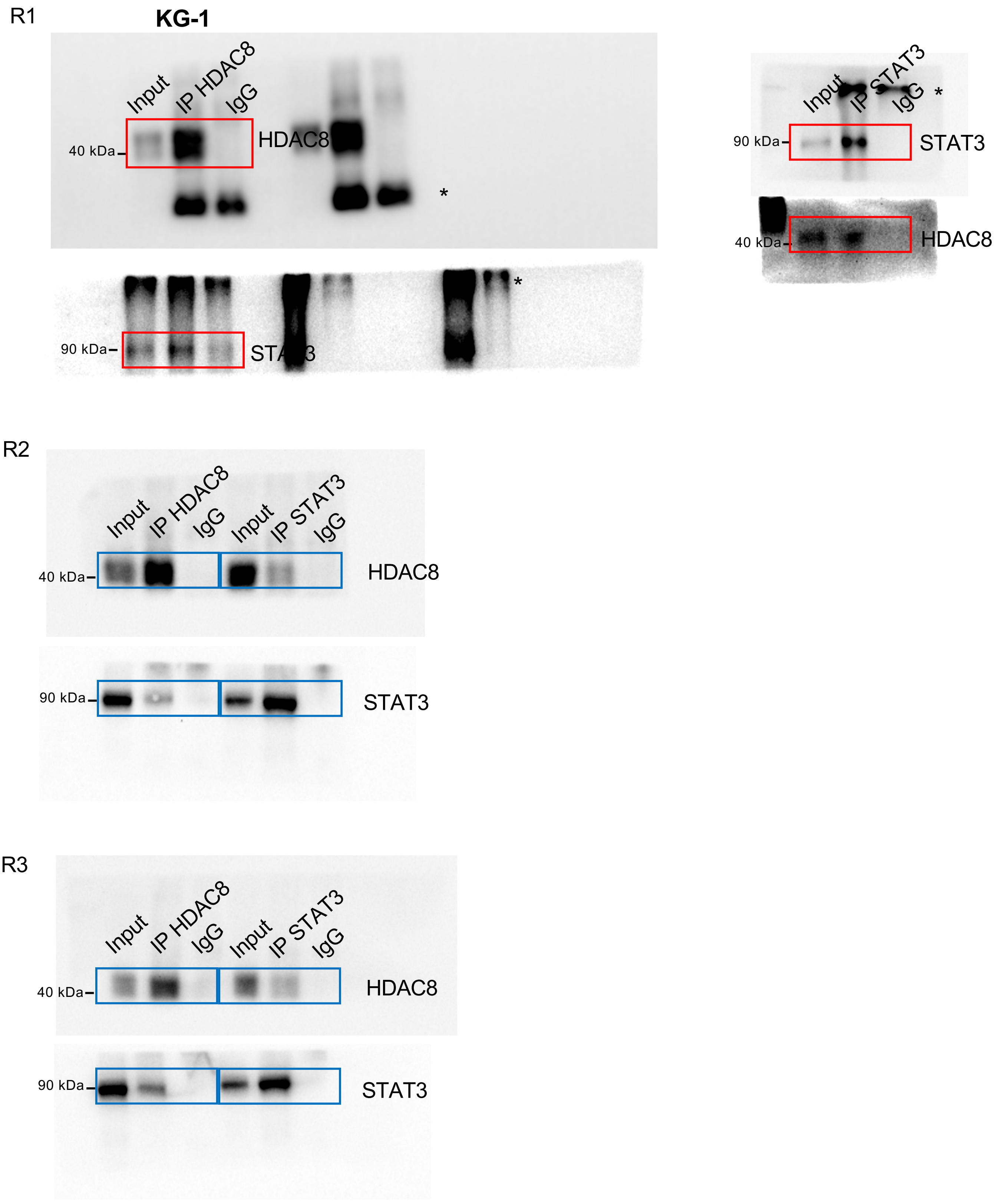

* non-specific band

Supportive Data

Figure S7B

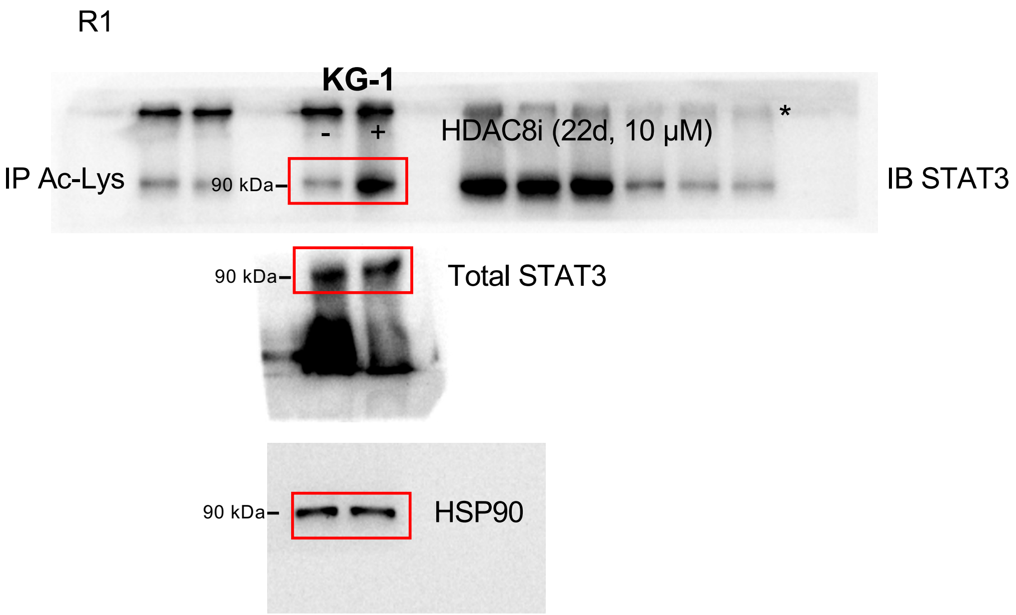

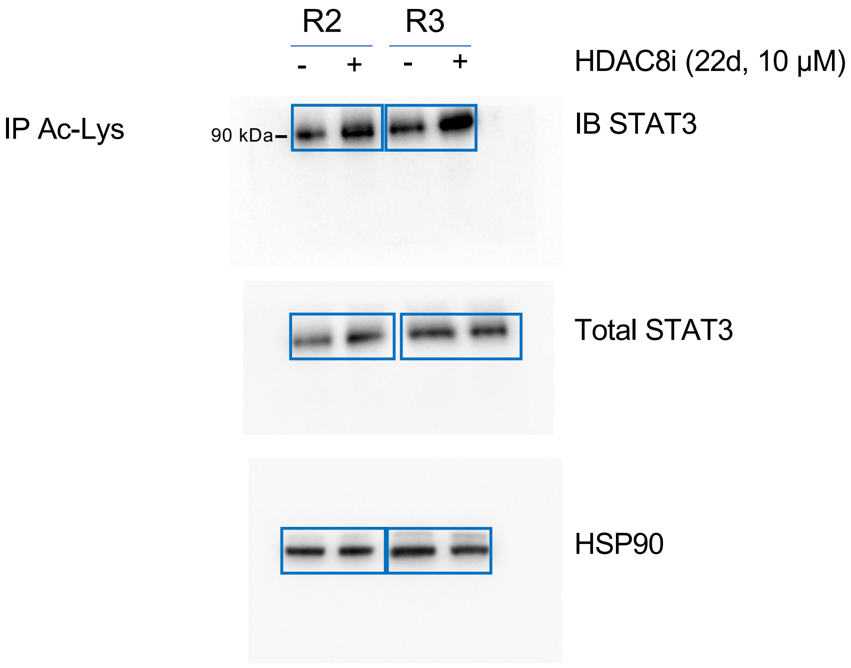

* non-specific band

Supportive Data

Figure S7D

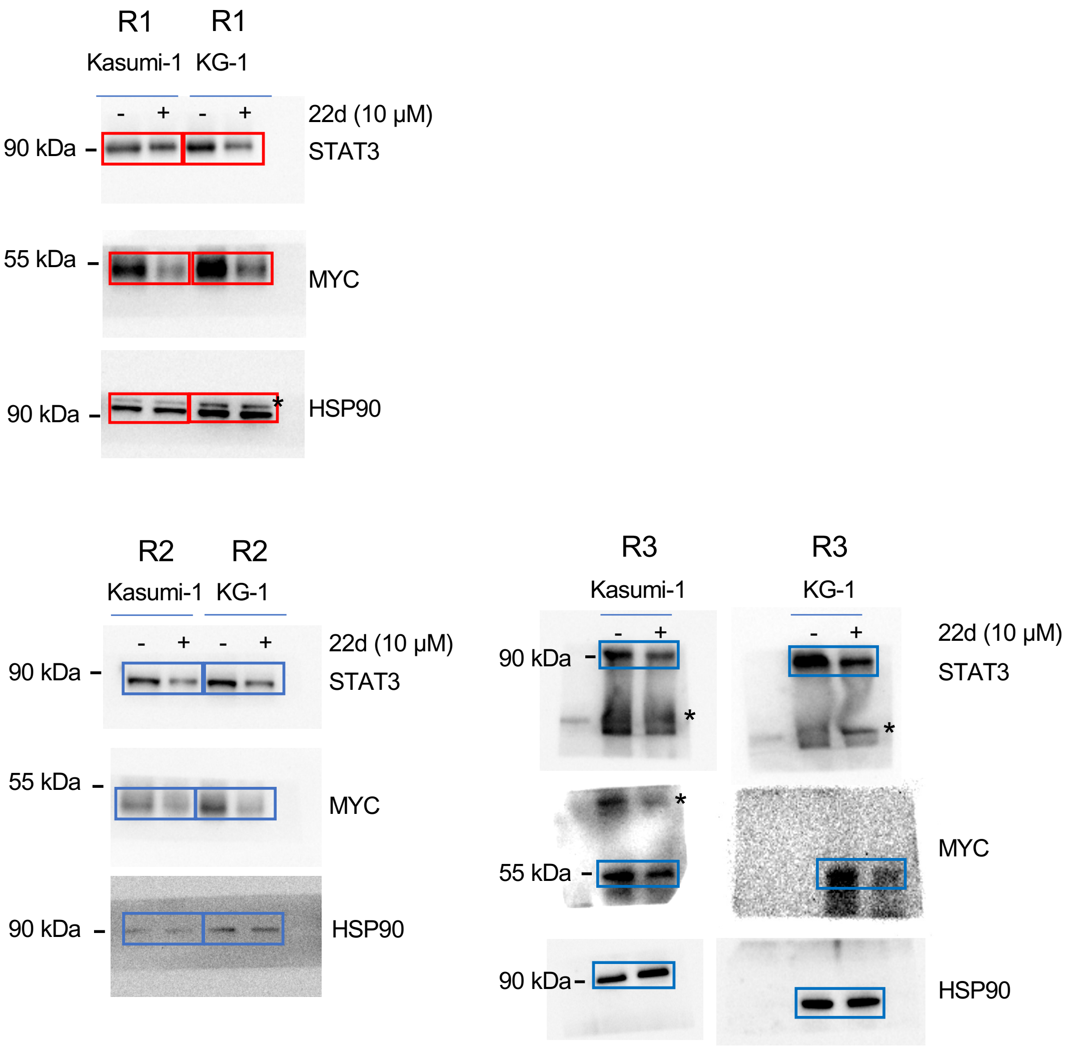

* non-specific band
